# Biochemical Characterization of a Family 15 Carbohydrate Esterase from a Bacterial Marine Arctic Metagenome

**DOI:** 10.1371/journal.pone.0159345

**Published:** 2016-07-19

**Authors:** Concetta De Santi, Nils Peder Willassen, Adele Williamson

**Affiliations:** The Norwegian Structural Biology Centre, Department of Chemistry, UiT—The Arctic University of Norway, Tromsø, Norway; INRA, FRANCE

## Abstract

**Background:**

The glucuronoyl esterase enzymes of wood-degrading fungi (Carbohydrate Esterase family 15; CE15) form part of the hemicellulolytic and cellulolytic enzyme systems that break down plant biomass, and have possible applications in biotechnology. Homologous enzymes are predicted in the genomes of several bacteria, however these have been much less studied than their fungal counterparts. Here we describe the recombinant production and biochemical characterization of a bacterial CE15 enzyme denoted MZ0003, which was identified by *in silico* screening of a prokaryotic metagenome library derived from marine Arctic sediment. MZ0003 has high similarity to several uncharacterized gene products of polysaccharide-degrading bacterial species, and phylogenetic analysis indicates a deep evolutionary split between these CE15s and fungal homologs.

**Results:**

MZ0003 appears to differ from previously-studied CE15s in some aspects. Some glucuronoyl esterase activity could be measured by qualitative thin-layer chromatography which confirms its assignment as a CE15, however MZ0003 can also hydrolyze a range of other esters, including *p-*nitrophenyl acetate, which is not acted upon by some fungal homologs. The structure of MZ0003 also appears to differ as it is predicted to have several large loop regions that are absent in previously studied CE15s, and a combination of homology-based modelling and site-directed mutagenesis indicate its catalytic residues deviate from the conserved Ser-His-Glu triad of many fungal CE15s. Taken together, these results indicate that potentially unexplored diversity exists among bacterial CE15s, and this may be accessed by investigation of the microbial metagenome. The combination of low activity on typical glucuronoyl esterase substrates, and the lack of glucuronic acid esters in the marine environment suggest that the physiological substrate of MZ0003 and its homologs is likely to be different from that of related fungal enzymes.

## Introduction

Glucuronoyl esterases (often denoted GEs, EC 3.1.1.-) are enzymes acting on the esters of 4-O-methyl-D-glucuronic acid (MeGlcA). These are designated as family *Carbohydrate Esterase 15* (CE15) in the Carbohydrate-Active enZYmes Database (CAZy; http://www.cazy.org/) and will be referred to here by this nomenclature. Phylogenetic analysis of the CE15s from fungi revealed this enzyme class forms a distinct clade apart from other carbohydrate esterases such as acetylesterases (EC 3.1.1.6), acetylxylan esterase (EC 3.1.1.72) and feruloyl esterases (EC 3.1.1.73). [1]. This is consistent with the unique substrate specificity of CE15s; the CE15 of *Schizophyllum commune* (*S*. *commune)* is active on esters of 4-O-methyl-alpha-D-glucopyranosyl uronic acid (MeGlcA) [2] and cannot de-esterify either methyl- or ethyl ferulate which are degraded by feruloyl esterases (EC 3.1.1.73), or *p-*nitrophenyl acetate (*p-*NP acetate) which is a common substrate used in generic esterase assays [2]. Conversely the MeGlcA-linked substrates methyl 4-O-methyl-D-glucopyranuronate and 4-nitrophenyl 2-O-(methyl 4-O-methyl-a-D-glucopyranosyluronate)-b-D-xylopyranoside are recalcitrant to the action of feruloyl esterases, acetylxylan esterases (EC 3.1.1.72) and pectin methylesterases (EC 3.1.1.11) [2]. Substrate recognition by CE15s involves the uronic acid part of the molecule rather than the alcohol; in particular, the 4-O-methyl substituent of MeGlcA is important for de-esterification and imparts a significant increase in catalytic efficiency when compared with the methyl derivative [3].

The biological substrates of CE15s studied to date are thought to be the ester linkages between uronic acid groups of the hemicellulose and the hydroxyls of lignin alcohols in the lignin-carbohydrate complexes (LCCs) of plant cell walls, thus these enzymes function to degrade plant material [2]. This proposed biological function is consistent with biochemical evidence of esterase activity on various substrate mimics of LCCs, and with the ability of some CE15s to efficiently cleave ester bonds between bulky substituents at either the MeGlcA part of the substrate or on the alcohol side of the ester [3, 4]. More recently, experiments with alkali extracted beechwood glucuronoxylan methyl ester provided the first evidence of de-esterification activity by CE15s on high molecular mass polysaccharides [5].

The majority of CE15s which have currently been characterized derive from saprotrophic fungi. The first-described CE15 was purified from spent culture supernatant of the white-rot fungus *S*. *commune* [2]. Subsequent to determination of the *S*. *commune* CE15 protein sequence, homology-based searches revealed that CE15s are found throughout the fungal kingdom, however they are neither limited to, nor ubiquitous in one fungal class. Fungal CE15s that have been studied include the *cip2* gene of *Hypocrea jecorina* (*H*. *jecorina; synonym Trichoderma reesei*), which was overexpressed in its native host prior to purification [6]; two isoforms from *Phanerochaete chrysosporium (P*. *chrysosporium)* denoted *ge1* and *ge2*, which were recombinantly expressed in both *Pycnoporus cinnabarinus (P*. *cinnabarinus)* and *S*. *commune* purified from the culture supernatant of its native host [1]; StGE2, also from *M*. *thermophile*, which was produced recombinantly from *Pichia pastoris (P*. *pastoris)* [7]. Recently two other novel fungal CE15s have been described; PaGE1 from *Podospora anserine* (*P*. *anserine*), which was functionally expressed in *P*. *pastoris* and the CuGE of *Cerrena unicolor (C*. *unicolor)* produced in *Aspergillus oryzae* [8, 9].

Much less research has focused on the CE15s of bacteria, despite many species having genes encoding homologs of the fungal enzymes. To date the activity of only one bacterial CE15 has been described *in vivo*; the C-terminal domain of the multi-domain CesA protein of *Ruminococcus flavefaciens (R*. *flavefaciens)* which was shown to de-esterify glucuronoxylan methyl ester as effectively as its fungal counterparts[5]. The N-terminal domain of CesA has separate acetyl xylan esterase activity, which has probably lead to many single-domain *ce15* genes being erroneously annotated as acetyl xylan esterases in bacteria [10]. Such genes are found in bacterial phyla Planctomycetaceae and Verrucomicrobia which are notoriously difficult to cultivate [11–13], suggesting that further diversity of bacterial CE15s may be accessible by cultivation-independent methods. In an attempt to exploit this potential source of variation, we have undertaken the recombinant expression, purification and characterization of a single-domain bacterial CE15 homolog, MZ0003, which derives from metagenomic bacterial DNA isolated from marine Arctic sediment. Here we report that MZ0003 has some glucuronoyl esterase activity; however it has a number of features that distinguish it from previously described CE15s and esters of glucuronic acid are probably not its biological substrate.

## Results

### Phylogeny analysis of MZ0003

*mz0003* originates from a metagenomic library prepared from sediment collected off the coast of Svalbard, and its product was initially annotated as an acetyl xylan esterase using the RAST Server, although no known catalytic domains were detected using Pfam [14–16]. MZ0003 appears to be a single domain protein with no dockerin or carbohydrate-binding modules predicted, while analysis using SignalP indicates a periplasmic-secretion leader peptide at the N-terminus [17]. Sequence homology searches of the Non-redundant UniProtKB/SwissProt sequences using BLAST found the greatest similarity between MZ0003 and CE15s; both fungal and CesA of *R*. *flavefaciens*. Subsequent searches against the Non-redundant protein sequences database identified many highly similar sequences from bacteria, most of which were annotated as *hypothetical* or *acetyl xylan esterase*.

**Fig 1:**
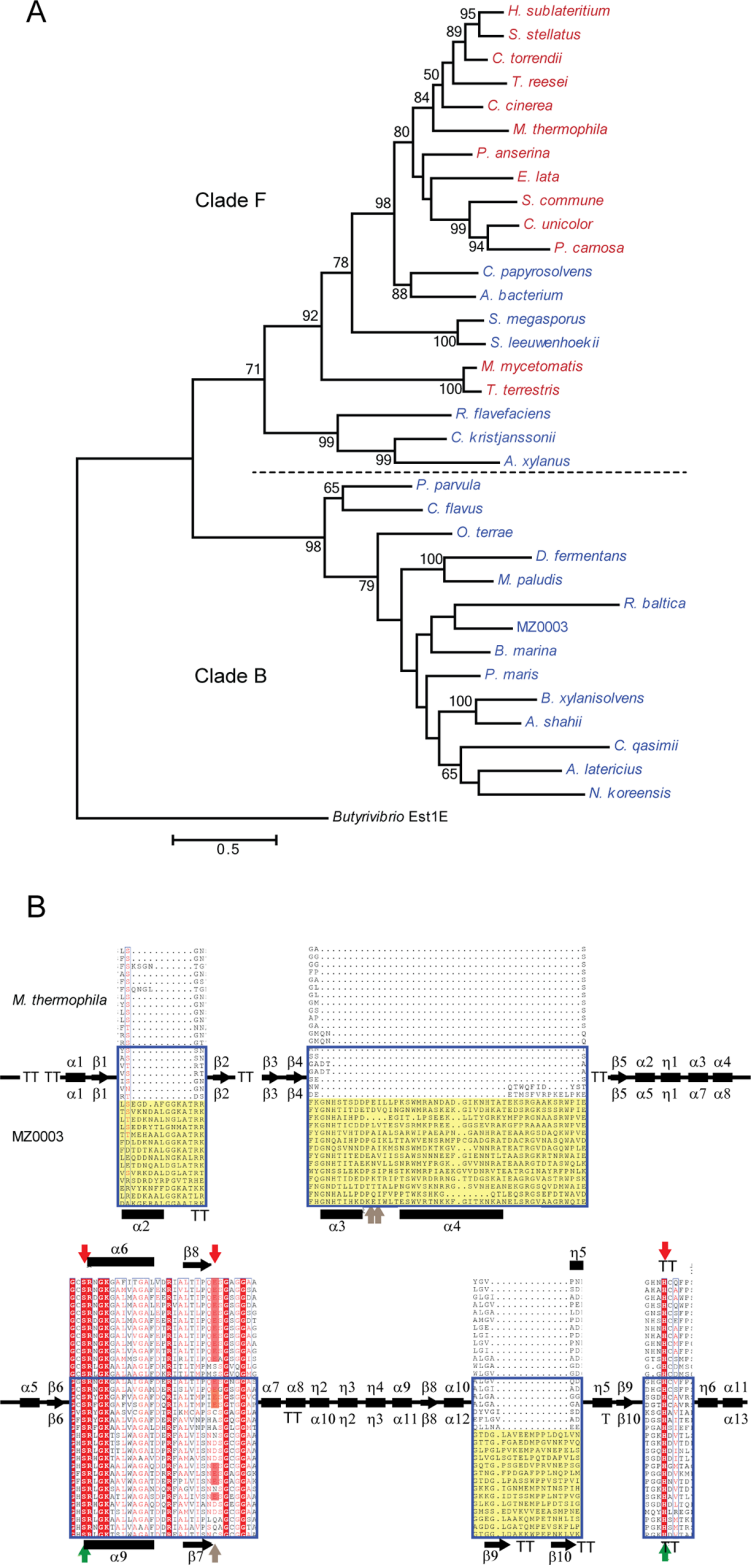
Phylogenetic tree and structural organisation of CE15s. A) Maximum Likelihood tree of CE15s from fungi (red text) and bacteria (blue text). A dashed line is used to highlight the deep-branching split between the two major clades, with ‘Clade F’ referring to previously-characterised fungal CE15s and their homologs, and ‘Clade B’ to MZ0003 and related bacterial sequences. The tree is drawn to scale, with branch lengths measured in the number of substitutions per site. Bootstrap values above 50% are shown. Evolutionary analyses were conducted in MEGA6. Full species name and gene identifiers of sequences are given in **S1B** Table) Schematic of the alignment of fungal and bacterial CE15 structures. Secondary structural elements from the *M*. *thermophilia* StGE2 sequence are shown with β-strands indicated by arrows, α-helices by oblongs and turns denoted by ‘TT’. Nomenclature for the StGE2 structure is given above, and the prediction for the MZ0003 structure below. Expanded areas 1–3 show the position of loops predicted in the MZ0003 model (discussed below) which are found in related bacterial homologs of ‘Clade B’. Bacterial sequences are indicated by the blue box, and putative loops are shaded yellow. Regions surrounding active site residues are also expanded, with fully conserved residues shaded red, and functionally conserved residues shown in red text. Confirmed active site residues of StGE2 are indicated by red arrows above the sequence, while catalytic residues identified by mutagenesis in MZ0003 are indicated in green on the lower sequence. Residues which were mutated without effect in MZ0003 are indicated with grey arrows. Details of the full sequence alignment are given in S1 Fig.

In an attempt to elucidate the possible taxonomic origin of MZ0003 and to explore the relationship between fungal and bacterial CE15s, a phylogenetic tree was built based on the alignment of the MZ0003 sequence with a selection of bacterial and fungal homologs that includes all CE15s characterized to date. CE15s form two deep-branching clades: the first, which includes all the fungal sequences and some bacteria, is termed ‘clade F’; the second, which includes exclusively bacterial representatives, is termed ‘clade B’. MZ0003 is placed in clade B closest to the three marine Planctomycetes species: *Blastopirellula marina* (*B*. *marina*) DSM 3645, *Planctomyces ma*r*is* (*P*. *ma*r*is*) DSM 8797 and *Rhodopirellula baltica* (*R*. *baltica*) SH 1 (Fig 1A). Inspection of the sequence alignment reveals that those bacterial sequences found in clade F, including CesA of *R*. *flavefaciens*, lack the large inserts identified in the MZ0003 sequence and other members of clade B (Fig 1B and S1 Fig). Another interesting feature of the alignment is that the catalytic Glu 249 of *M*. *thermophilia* StGE2 is not conserved among all sequences, being replaced with either Asp, Gln, Asn, Ser, Ala or as in the case of MZ0003, Cys (S1 Fig). The consensus sequence G-C-S-R-x-G, which contains the catalytic serine and is characteristic of CE15s [7] was essentially preserved in bacterial sequences with the exception of the cysteine in the second position which in some species was replaced by His, Phe or in two cases Gly or Val.

### Production of recombinant MZ0003

MZ0003 was expressed in *E*. *coli* from pCold II as described previously [18], and the resulting soluble protein was purified to homogeneity as judged by SDS-PAGE (S2A Fig) which confirmed a molecular weight of 45.7 kDa. SDS-PAGE in the absence of reducing agent indicates there are no intermolecular disulfide bonds, however higher molecular weights of 61.2 kDa and 66.0 kDa were predicted by gel filtration and native PAGE respectively, indicating the recombinant protein is monomeric and may adopt an extended conformation in solution (S2B Fig).

### Esterase activity of MZ0003: Substrate specificity and kinetics

**Fig 2:**
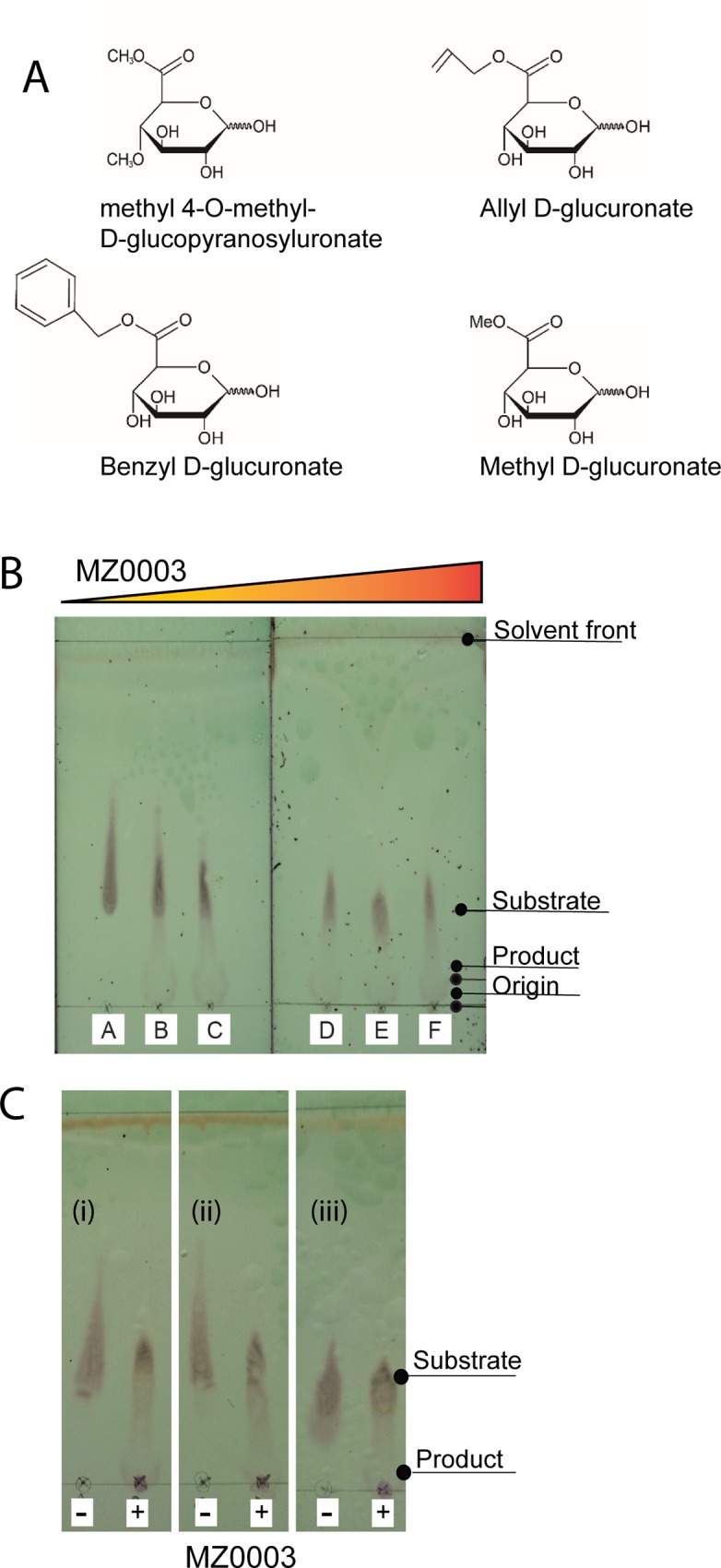
Specific activity of MZ0003 measured by TLC. A) Substrates used to detect glucuronoyl esterase activity by TLC. **B)** De-esterification of methyl 4-O-methyl-D-glucopyranuronate. MZ0003 concentrations used in the assay are: (A) 0 μg, (B) 5 μg, (C) 10 μg, (D) 15 μg, (E) 25 μg, (F) 50 μg in a final volume of 100 μl. **C)** De-esterification of allyl D-glucuronate (i), benzyl D-glucuronate (ii) and D-glucuronic acid methyl ester (iii) by 50 μg MZ0003 (+), or incubated in the absence of enzyme (-). Activity was measured after 90 minutes at 25°C for all substrates.

MZ0003 demonstrated some activity in a qualitative TLC-based assay for de-esterification of methyl 4-O-methyl-D-glucopyranosyluronate (Fig 2A), a substrate which has been used extensively to assay for glucuronoyl esterase activity ([1, 2, 7, 19, 20](Fig 2B). Activity against three other substrates which lack the 4-O-methyl substituent, allyl D glucuronate, benzyl D-glucuronate and D-glucuronic acid methyl ester (Fig 2A) was also detected indicating that MZ0003 does not require this for its activity, and that, in the case of benzyl D-glucuronate it can hydrolyze glucuronic acid esters with bulky substituents on the alcohol side (Fig 2C). In all cases hydrolysis appeared to be very low suggesting that although MZ0003 has glucuronoyl esterase activity, this is probably not its native or biological substrate. To indicate whether hydrolysis by MZ0003 was the result of general esterase activity, methyl 4-O-methyl-D-glucopyranosyluronate was incubated with the carboxylesterase ThaEst2349 [21] under the same conditions; however no product was observed with this enzyme even after 18 hours (S3 Fig).

To test the range of substrates tolerated by MZ0003 its activity on a variety of esters, other than glucuronic acid derivatives, was determined using quantitative assays. The highest specific activity was found with the small acetyl ester *p-*NP acetate, which was measured by both time-resolved and endpoint assays, as described in *Materials and methods* to allow direct comparison with other substrates (Table 1). The relative MZ0003 activity ranged between 11–18% with 4-methylumbelliferyl acetate, α-naphtyl acetate, 5-bromo-4-chloro-3-indoyl acetate and β-D-galactose pentaacetate and 2–8%, when 7-aminocephalosporanic acid, β-D-glucose pentaacetate and cellulose acetate were used, while no esterase activity was detected against *p*-NP octanoate (Table 1).

**Table 1:** Substrate specificity of MZ0003 towards *p*-NP esters and acetyl esters. **a:** Rate was measured using the time-resolved spectrophotometric assay for detection of liberated *p*-nitrophenol group expressed per mg of protein. **b**: Activity was measured as the relative amount of acetyl group liberated per mg of protein by endpoint assay. Activities with each substrate are expressed relative to the activity with *p-*NP acetate, which was measured using both methods. All measurements were done at 35°C and represent the mean of three independent determinations with the standard deviation given as the error.

Substrate	Relative Esterase Activity (%)
*p-*NP acetate ^a, b^	100.0 ±1.0
5-Bromo-4-Chloro-3-Indoyl acetate^b^	18.4 ±3.0
β-D-Galactose pentaacetate^b^	15.0 ±5.0
4-Methylumbelliferyl acetate^b^	14.0 ±5.0
1-Naphtyl acetate^b^	11.7 ±2.8
*p-*NP butyrate^a^	10.0 ±4.0
β-D-Glucose pentaacetate^b^	8.0 ±1.6
Cellulose acetate^b^	2.8 ±0.3
7-Aminocephalosporanic acid^b^	2.3 ±0.5
*p-*NP octanoate^a^	0

Determination of *p*-NP acetate hydrolysis by the time-resolved assay was also used to measure the kinetics of MZ0003 which hydrolyzed it with a turnover Kcat/Km = 12.5 ± 2.3 s^-1^ mM^-1^ in the absence of NaCl, and Kcat/Km = 13.2 ± 2.4 s^-1^ mM^-1^ with 1M NaCl (S4 Fig). This substrate was used for further characterization of MZ0003 as it provides a convenient direct method to measure MZ0003 activity under various conditions.

### Temperature optimum and thermal stability

**Fig 3:**
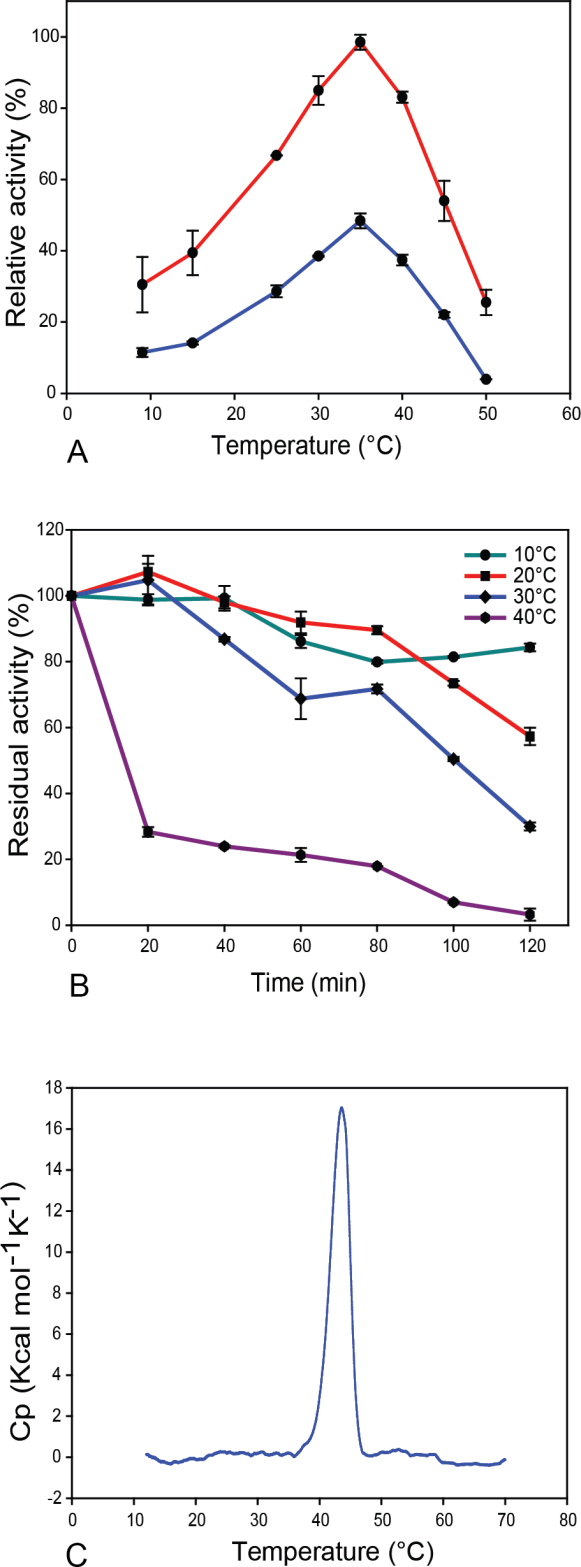
**Temperature dependence of activity and stability of MZ0003 A)** Temperature optimum: activity represents the initial rate of the reaction at each temperature in the presence of 1 M NaCl (upper plot) and no added salt (lower plot), normalised to the highest rate, which was recorded at 35°C in 1 M NaCl. **B)** Temperature stability: residual activity represents the initial rate of the reaction measured at 35°C after incubation for various lengths of time at the temperatures shown. Values are normalised to the rate recorded without pre-incubation. All data points for assays represent the mean of three independent experiments, and error is given as the standard deviation. **C)** Thermal unfolding measured by DSC: representative thermogram with buffer subtracted and a sigmoidal baseline fitted.

The optimal temperature of MZ0003 acting on *p*-NP acetate was 35°C, above which activity significantly decreased, while 30% of the maximum activity was recorded at 10°C (Fig 3A). Inclusion of 1 M NaCl lead to a consistent increase in relative activity of approximately 50% across all temperatures. The thermal stability of MZ0003 was examined by measuring its residual activity after incubation at various temperatures over a period of 120 min. At 10°C almost 80% of the original activity was retained, while at 20°C and 30°C the residual activity declined over the entire time course. A significant loss in activity was detected at 40°C with less than 30% remaining after 20 min, and complete inactivation occurring between 100 and 120 min (Fig 3B). The temperature optimum of activity and stability are consistent with the thermal stability of MZ0003 measured by differential scanning calorimetry (DSC). The thermogram indicates a single unfolding transition occurring between 35°C and 48°C with a T_m_ of 43.3°C, a calorimetric enthalpy change (∆H_cal_) of 74.36 Kcal mol^-1^ and an entropy change (∆S) of 0.23 Kcal mol^-1^ K^-1^ (Fig 3C). Fitting of a two-state scaled model calculated a van't Hoff enthalpy change (∆H_vH_) of 202.24 Kcal mol^-1^ indicating that unfolding of MZ0003 is not a two-state transition as the ratio of ∆H_vH_ to ∆H_cal_ is significantly greater than 1; however because denaturation of MZ0003 was irreversible and therefore kinetically driven, it was not possible to determine whether this deviation was due to cooperative unfolding or aggregation processes, and the thermodynamics of unfolding were not investigated further. No exothermic aggregation signals were observed in the raw data (not shown). These data indicate that despite its marine Arctic origin, MZ0003 is functional at temperatures comparable to mesophilic enzymes [22].

### MZ0003 activity optima: pH, salt and tolerance for inhibitors

**Fig 4:**
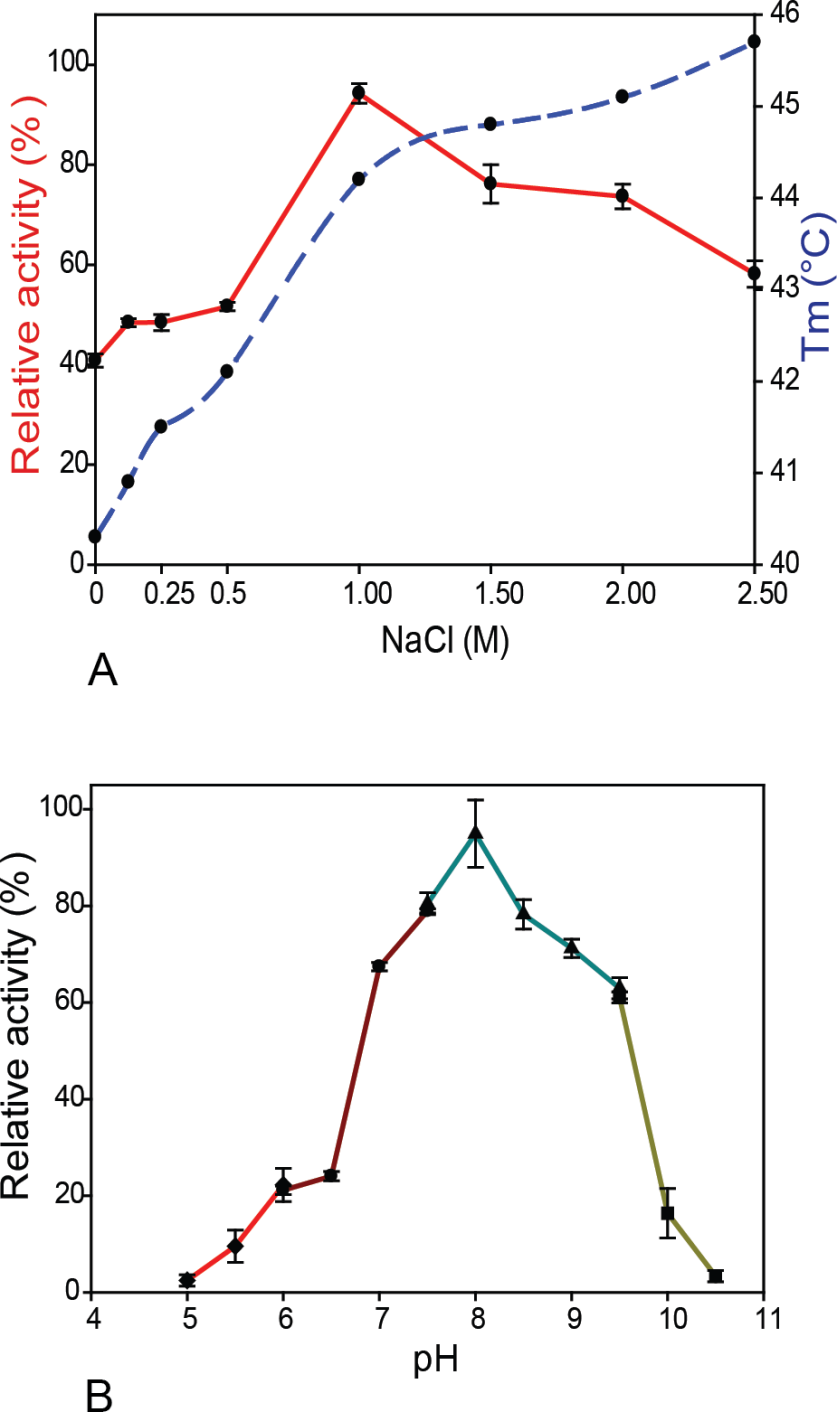
The effect of salt and pH on MZ0003. **A)** Thermal stability (blue dashed line) and activity (red solid line) with varying NaCl concentrations. Stability was determined by DSF in 50 mM HEPES pH 8.0, activity was tested in 0.1 M Tris-HCl pH 8.0 and defined as the percentage relative to the maximum enzyme activity **B)** Effect of pH on MZ0003 tested at 30°C: MES was used for pH 5.0–6.0, Na-phosphate buffer from pH 6.0–7.5, Tris-HCl buffer for pH 7.5–9.5 and CAPS buffer for pH 9.5–10.5. All data points for assays represent the mean of three separate experiments.

The marine source of the *mz0003* gene isolated from the Barents Sea where the salinity is 33–35 psu (corresponding to approximately 0.6 M NaCl), coupled with its predicted periplasmic location suggests it may carry out its biological function under high salt concentrations, thus enzyme activity on the substrate *p*-NP acetate was determined at NaCl concentrations from 0 to 2.5 M. The highest esterase activity was observed at 1 M NaCl, which declined to 60% at the maximum salinity tested, while in the absence of NaCl, MZ0003 retained only 40% of its activity (Fig 4A). These activity data correlate well with the increasing stability measured by differential scanning fluorimetry (DSF) between 0 and 1 M NaCl although the greater thermal stability at increased NaCl concentrations does not produce an equivalent increase in activity (Fig 4A). MZ0003 showed more than 60% of its activity in the pH range of 7.0–9.5, with an optimal pH 8.0 in Tris-HCl buffer. Twenty percent of the activity was retained at low pH range and at pH 10.5, while no catalytic activity was found at pH 5.0 or 10.5 (Fig 4B). The relative pH-dependent activity profile was essentially unchanged by the inclusion of 1M NaCl in the buffer.

**Fig 5:**
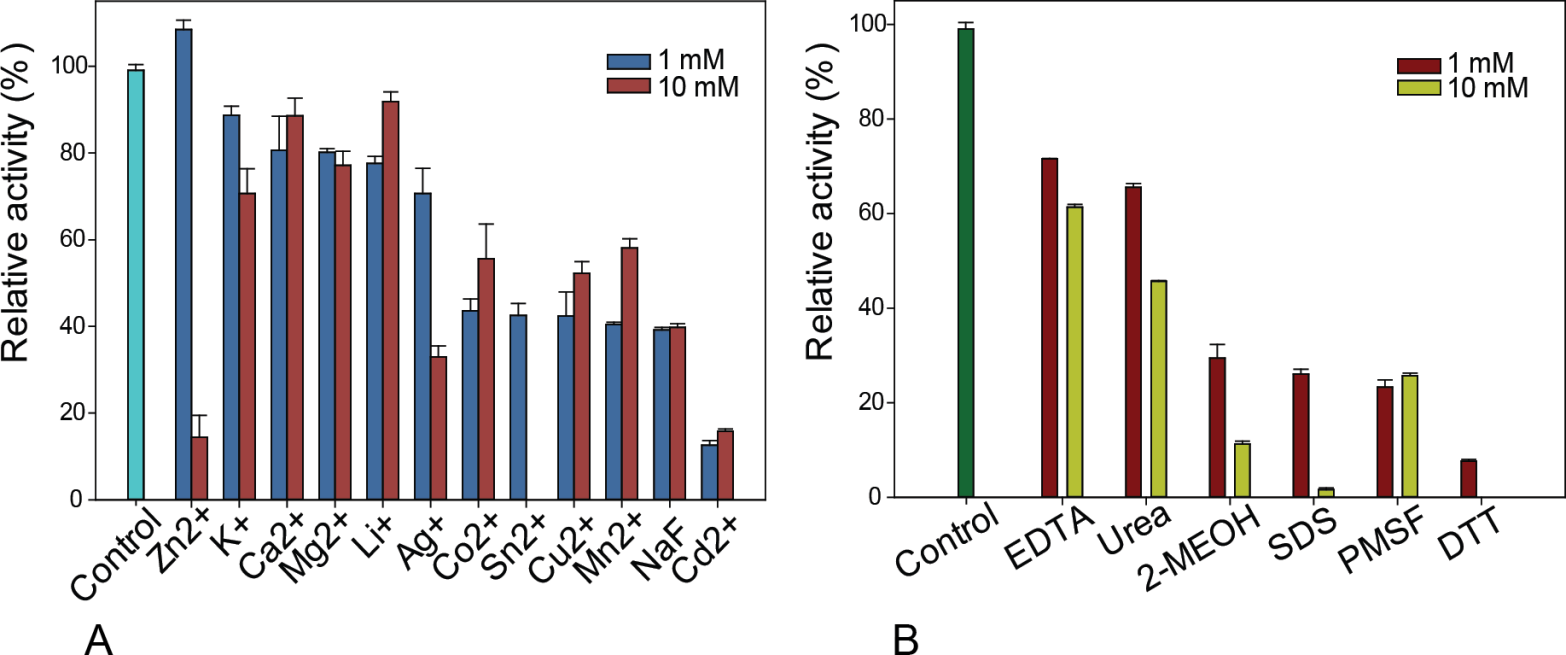
Effect of various metal ions, common inhibitors and denaturants on MZ0003 activity. **A)** Activity tested with addition of KCl, LiCl, AgCl_2_, NaF, ZnCl_2_, CaCl_2_, MgCl_2_, CoCl2, SnCl_2_, CuCl_2_, MnCl_2_ and CdC_l2_ at concentrations of 1 and 10 mM. **B)** Activity tested with: EDTA, Urea, 2-Mercaptoethanol (2-MeOH), SDS, PMSF and DTT at concentrations of 1 and 10 mM. MZ0003 was incubated with each chemical for 1 hour in 0.1 M TRIS HCl pH 8.0 on ice and then the activity was tested with p-NP acetate at 35°C. All data are the average of three independent experiments, error is the standard deviation.

To determine whether MZ0003 required any metal cofactor for catalysis, or conversely whether these and other chemical reagents were detrimental to its activity, MZ0003 was assayed in the presence of various metal ions, common inhibitors and denaturants (Fig 5). None of the 12 metal additives tested gave any significant stimulation of activity, while even very high concentrations of EDTA produced only a moderate decrease in activity, suggesting that metal ions are not required. A significant reduction of activity of MZ0003 was observed with 10 mM of Zn^2+^ as well as with Cd^2+^ at both 1 and 10 mM. Co^2+^, Sn^2+^, Cu^2+^ and Mn^2+^ decreased activity by between 30–40%. Inhibition by metal ions may be due to their non-specific interactions with the enzyme as has been previously reported [23]. Inhibition of 80% of MZ0003 activity by 1–10 mM PMSF and 60% by NaF is consistent with the catalytic nucleophile being a Serine. The chaotropic denaturant urea decreased activity by 40%, and the detergent SDS by over 60% even at relatively low concentrations, which is consistent with MZ0003 having a low overall stability. Inhibition by reductants DTT and 2-MeOH may indicate the presence of disulfide bonds in the MZ0003 structure.

In summary, MZ0003 appears to be a salt-tolerant enzyme, which is active at moderate pH and does not require metal ions for activity.

### Structural modelling and Site-directed mutagenesis

To gain insight into the structure of MZ0003, a homology model was made based on the structurally characterized *M*. *thermophilia* CE15 enzyme StGE2 (PDB 4G4G). Currently structures are available for two CE15s: the catalytic domain of Cip2 GE from *H*. *jecorina* and the full-length StGE2 [24, 25]. Both enzymes comprise a three-layer αβα-sandwich typical of serine-type hydrolases and both produced very similar predictions for the structure of MZ0003; however, StGE2 was chosen due to its higher resolution and because the structure of an active site mutant of this enzyme has been determined in complex with the 4-O-methyl-D-glucopyranosyluronate substrate.

**Fig 6:**
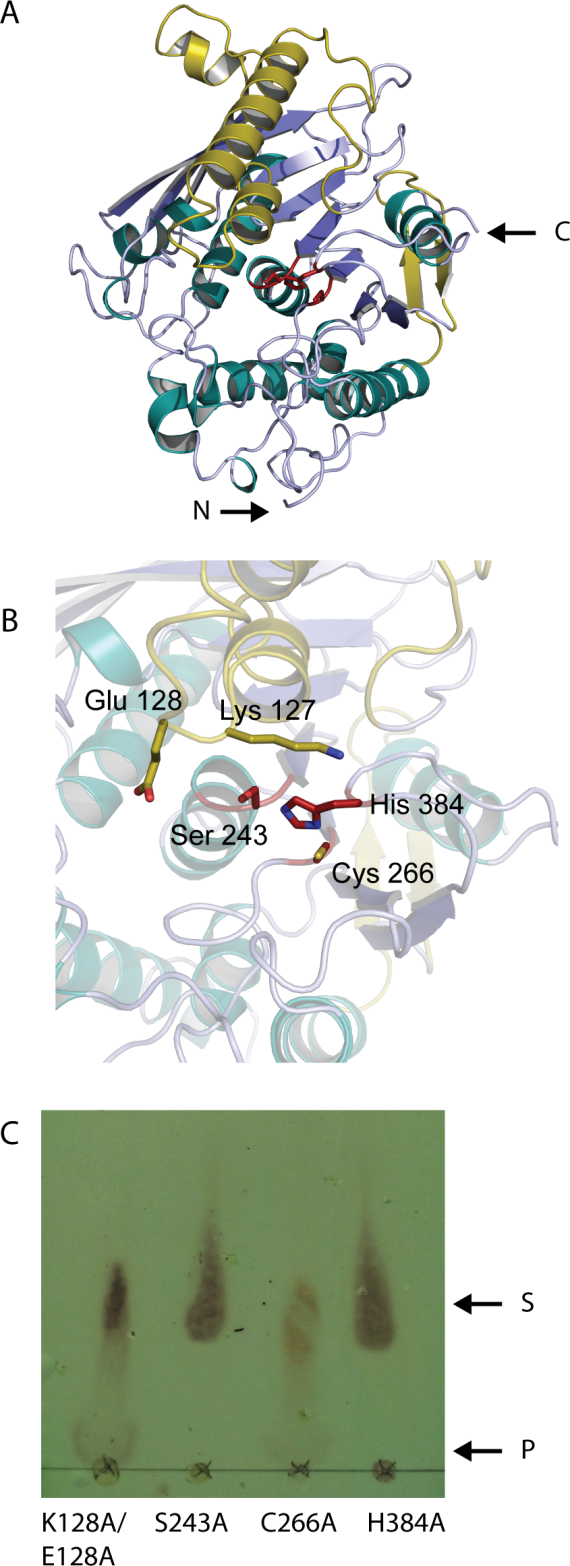
Structural model of MZ0003 and mutagenesis. **A)** Overall structure of MZ0003: α-helix of regions that aligned with the M. *thermophilia* StGE2 sequence are shown in cyan, β-strands in violet. Large regions, which could not be aligned based on sequence, are shown in yellow. **B)** Predicted active site of MZ0003 studied by site directed mutagenesis: residues in equivalent positions to the catalytic triad of StGE2 are shown as red sticks. Nearby polar residues in the predicted loop are shown as yellow sticks. **C)** Esterase activity of mutants on methyl 4-O-methyl-D-glucopyranosyluronate at 25°C measured after 90 minutes by TLC. Activity seen as the appearance of the lower product spot (indicated by P) and the decrease in intensity of the upper substrate spot (S).

The MZ0003 model predicts three large insertions: 15 residues which form a loop and helix between β1 and β2, 47 residues between β4 and β5 which form two helices, and 18 residues which form a β hairpin before the 8th β-strand of the core β-sheet (Fig 6A). The presence of these regions, which correspond to the insertion in sequences of the ‘clade B’ group of the phylogenetic tree (Fig 1) have no counterpart in the StGE2 structure based on sequence alignment. This probably explains the poor overall modelling score of the MZ0003 model (Global Model Quality Estimation of 0.5) as these parts of the model have local quality estimates significantly below this value. Excluding these regions, the majority of MZ0003, including the active site residues, was modelled with reasonable confidence (local Qmean scores: Ser 243, 0.724804; His 384, 0.587571; Cys 266, 0.745942, S5 Fig).

Analysis of the residues in equivalent positions to the StGE2 catalytic triad identified Ser 243 as the catalytic nucleophile of MZ0003, and His 384 as the base adjusting its nucleophilic character (Fig 6B). The third member of the triad is typically an acidic moiety, Glu 249 in StGE2; however the equivalent position of the MZ0003 model is occupied by Cys 266, which cannot fulfill the same chemical function. Putative residues involved in the catalytic triad or in the selectivity of the substrate were mutated to alanine to generate four mutants, which were tested for activity using standard conditions. Mutation of either Ser 243 or His 384 to alanine completely abolished esterase activity of MZ0003 with both the *p*-NP acetate and methyl 4-O-methyl-D-glucopyranosyluronate substrates, confirming their involvement in catalysis (Fig 6C and Table 2). Mutation of Cys 266 had no effect on activity indicating that it is not involved in catalysis, nor was it structurally necessary for the active conformation of the enzyme. A double mutant was also made of Lys 127 and Glu 128, which are positioned at the entrance to the catalytic site in the structural model; however this had no effect on the activity with either substrate. The stability of all four mutants with and without 1 M NaCl was analysed by DSF and gave T_m_ values identical to the wild type, confirming that inactivity in the S243A and H384A mutants was not due to denaturation of the overall protein structure and also suggesting that Cys 266 is not essential for MZ0003 stability, for example by participation in a disufide bond.

**Table 2:** Specific activity of mutants of MZ0003 acting on *p-*NP acetate. Activity is expressed as the μM *p*-NP liberated per mg protein under standard conditions. Measurements represent the mean of three independent determinations with standard deviation given as the error.

Mutant	Specific activity (μM *p-*NP mg ^-1^)
WT	13.7± 0.12
S243A	na
H384A	na
C266A	13.4 ± 0.09
K127A/ E128A	14.0 ± 0.18

na: no activity

## Discussion

### Taxonomic placement and putative function of MZ0003

MZ0003 is the second CE15 of bacterial origin which is confirmed to have glucuronyl esterase activity, and the first characterized single-domain CE15 from bacteria, as CesA from *R*. *flavefaciens* has an N-terminal acetyl xylan esterase catalytic domain in addition to the C-terminal CE15. Phylogenetic studies indicate that MZ0003 diverges significantly from CesA, having the highest identity to CE15s from plantomycetales *B*. *marina* (54% identity) *P*. *maris* (57% identity) *R*. *baltica* (46% identity).

The phyla Planctomycetaceae, Bacteroidetes and Verrucomicrobia, which contain CE15s that are the most similar to MZ0003, include species that are known to use carbohydrates as energy sources, are widespread in the marine environment and have been implicated in carbohydrate degradation in marine Arctic environments [11, 13, 26, 27]. These taxonomic groups are difficult to cultivate, and much of the information about the metabolic capabilities of these bacteria comes from metagenomic studies or functional studies of enzyme activities in intact microbial communities from raw isolates [11, 12, 28, 29]. It is very unlikely that the CE15 enzymes of these marine bacteria function in lignin degradation, as has been proposed for the fungal homologs [2, 8, 19], due to the presumably low content of plant secondary cell wall material in the ocean. The presence of wood-degrading enzymes has been documented in a few marine bacteria; a recent study examining lignin degradation by bacterial strains deriving from deep sea sediments confirmed the presence of enzymes able to cleave the β-O-4 linkage of lignin analog substrates [30]. Meanwhile the genome of the gamma proteobacterium *Teredinibacter turnerae*, an intracellular endosymbiont of wood-boring shipworms, is rich in enzymes degrading complex polysaccharides and includes a putatively multifunctional enzyme TERTU_3447 with a CE15 domain coupled to an N-terminal GH11 domain in the same reading frame [31]. However more likely biologically-relevant substrates for MZ0003 and other marine bacterial CE15s are algal cell wall polymers, as these would be readily available in this environment. The marine flavobacterium *Zobellia galactanivorans* (*Z*. *galactanivorans*) is able to use alginate as a sole carbon source and has two alginate degrading operons in its genome as well as several isolated alginolytic genes [32]. *Z*. *galactanivorans* also contains a single-domain CE15 enzyme with 50% identity to MZ0003.

**Fig 7:**
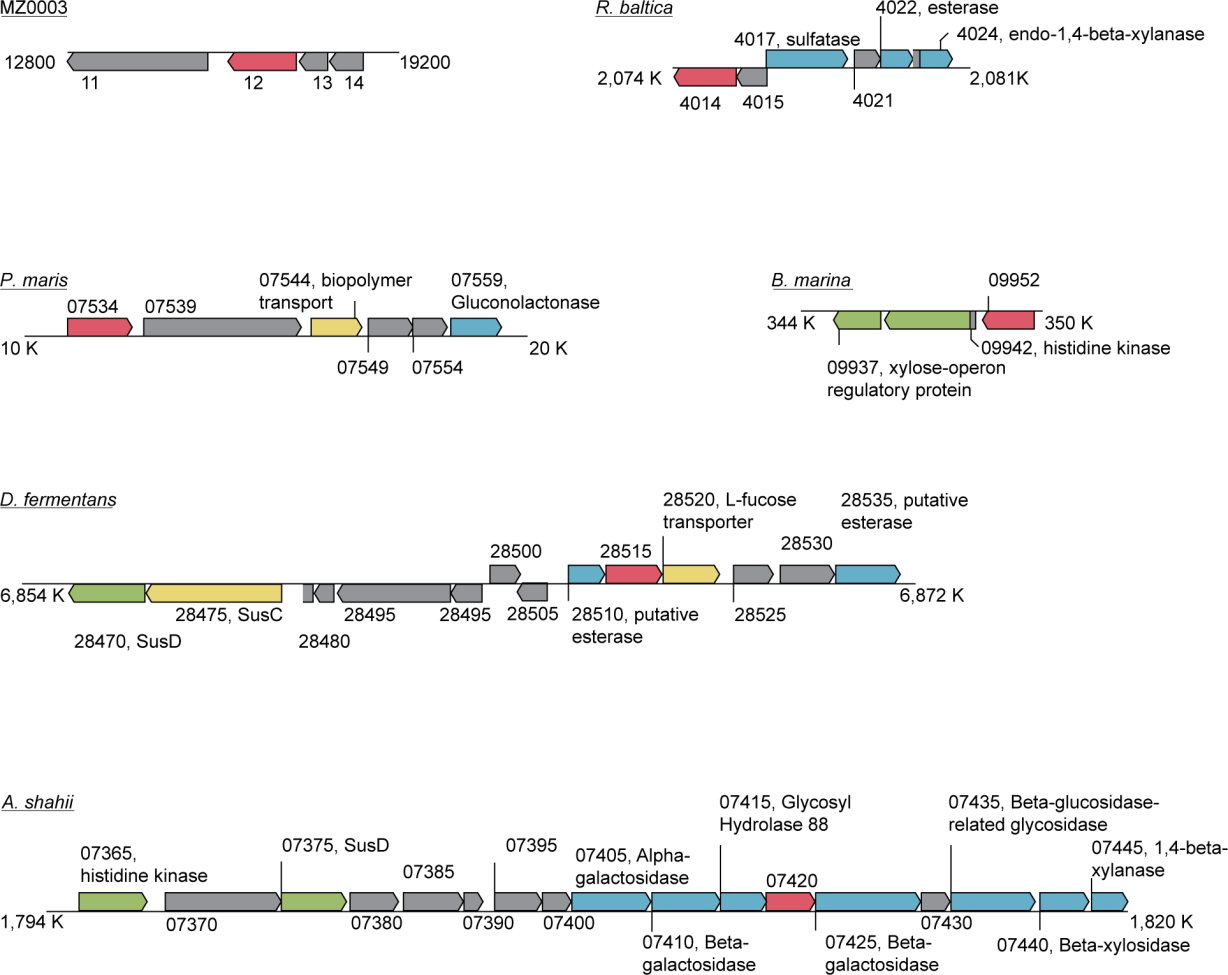
Organisation of genes surrounding the CE15 homolog which have functions that may be linked to carbohydrate degradation. Homologs of MZ0003 are coloured red, genes annotated as carbohydrate degrading enzymes are coloured blue, transporters are coloured yellow and regulatory proteins green. Hypothetical proteins and those with unrelated functions are shown in grey. The figure was produced manually based on graphics from the Artemis genome viewer.

To determine the possible biological roles of CE15s, the putative function and synteny of genes surrounding *mz0003* homologs were investigated for several bacteria from clade B of the CE15 phylogenetic tree (Fig 1). Although the specific arrangement of genes differed between species, all had orientations that indicated the *mz0003* homolog is part of an operon and many of the flanking genes have functions in carbohydrate degradation and transportation (Fig 7). The presence of the SusD/SusC sensor/transporter pairs in *Alistipes shahii* (*A*. *shahii)* and *Dyadobacter fermentans (D*. *fermentans)* suggest the CE15 enzyme is part of a polysaccharide utilization loci in both Bacteriodetes species. Our attempts to ascribe a biological function to MZ0003 by consideration of its genomic context were frustrated by the lack of annotation of flanking genes found in the fosmid contig, which is consistent with reports that gene prediction for several Planctomycetaceae genomes could only assign functions to around half the open reading frames [33].

### Structural and activity aspects of MZ0003

There are several ways in which the structure and function of MZ0003 appears to differ from previously characterized CE15s, despite them being similar at a sequence level and apparently having a shared evolutionary history.

MZ0003 and other members of clade B of the CE15 phylogenetic tree have large regions of primary sequence that are not found in members of clade F, which includes all characterized CE15s (Fig 1). The placement of these regions in the multiple sequence alignment together with a homology model of MZ0003 indicates they form loops that could be positioned at the entrance of the active site and play a role in substrate binding. Mutation of two large charged residues Glu 128 and Lys 127 had no effect on MZ0003 activity, however as these loops represent the poorest quality regions of the MZ0003 model, there is considerable uncertainty about the real orientation of these side chains relative to the active site (Fig 6). The structure of these putative loops and their possible role in substrate binding would be clarified by obtaining a high-resolution structure of MZ0003 or a close homolog, but it is plausible that they would contribute to differences in substrate specificity and activity compared with CE15s of fungal origin.

Inspection of the multiple sequence alignments between various CE15s, coupled with homology modeling of MZ0003 and mutagenesis studies pointed to further differences in the active sites of these enzymes. Structural modeling of MZ0003 surprisingly indicated that a cysteine is found in the position occupied by an acidic moiety of the catalytic triad in *M*. *thermophilia* StGE2. Mutagenesis of Cys 266 refuted any involvement in esterase activity, either catalytic or structural, and the multiple sequence alignment indicates that the third glutamic acid is not fully conserved among either bacteria or fungi. The most likely explanation is that another proximal acidic residue provides this function in these species, although there are examples of carbohydrate esterases that use only a Ser-His catalytic dyad such as the CE2 acetyl xylan esterases [34]. Again, a high-resolution structure of MZ0003 would be informative in suggesting alternative acidic residues as a third member of the catalytic triad.

Enzymatic assays of MZ0003 on various ester substrates also indicated it has a range of activities that differ from some previously characterized CE15s. As with fungal homologs, glucuronoyl esterase activity of MZ0003 could be qualitatively demonstrated using the substrate methyl 4-O-methyl-D-glucopyranosyluronate, albeit at a low level; however unlike the CE15 of *S*. *commune* [2], MZ0003 was also able to de-esterify the commonly-used esterase substrate *p-*NP acetate. The broader substrate scope of MZ0003 esterase activity and qualitatively low hydrolysis of esters of glucuronic acid derivatives suggests that this may represent an off-target or promiscuous activity which would be consistent with the lignin-hemicellulose ester bond not being its physiological substrate as discussed above. A fuller picture of the substrate specificity of MZ0003 will be gained by testing with a broader range of MeGlcA ester substrates, as well as examining activity on possible biologically relevant substrates such as polysaccharide components of algal cell walls.

Much of the research into fungal CE15 enzymes has focused on their possible application in pre-treatment of lignocellulosic material for conversion of plant biomass to fuel, or in the pulp and paper industry based upon their de-esterification activities on substrate-mimics of ester bonds in of LCCs [4]. The possible utility of MZ0003 in such processes must await demonstration of its activity on substrate analogs of lignin-carbohydrate complexes, however the broader or more promiscuous activity profile of this enzyme relative to fungal CEs suggests possible applications in degradation of other biological materials; particularly as the native activity of MZ0003 is unlikely to be lignin-hemicelluose ester bonds. As we speculate that the biological substrate for MZ0003 may be some component of algal polysaccharides, MZ0003 may be useful in purification or modification of these polymers, some of which have bioactive properties that make them attractive as potential drugs or as ‘functional foods’ [35, 36]. Traditional approaches for extraction use mechanical and chemical treatments to release water-soluble carbohydrate components, however depolymerizing enzymes may provide a milder alternative [37, 38]. In this regard, identification of psychrophilic homologs to MZ0003 would be particularly attractive, as extraction under low temperatures conditions would be expected to preserve integrity of the product [39].

To conclude, this study suggests that the bacterial CE15 MZ0003 differs from previously studied homologs in aspects of its activity and structure, which indicates the potential diversity of this class of enzyme in prokaryotes. As many of the most similar sequences to MZ0003 derive from difficult to culture bacterial phyla, investigation of the marine microbial metagenome may provide a route to further explore the potential of these enzymes.

## Materials and methods

### Phylogenetic and genome context analysis

The full length MZ0003 was used as a query sequence against the Non-redundant UniProtKB/SwissProt sequences and NCBI Non-redundant protein sequences databases using the BLASTP algorithm with default parameters (May 2015). The taxonomy report based on the latter analysis identified homologs in 137 bacterial species with highest scores from the phyla Planctomycetaceae, Bacteroidetes and Verrucomicrobia. The majority of these proteins were annotated as either acetyl xylan esterases or hypothetical products, and bacterial representatives from this set were included in the tree. Additional sequences including both fungal and bacterial proteins were chosen from a search using the product of *R*. *flavefaciens* gi|5834678 as a query sequence.

An initial multiple sequence alignment was constructed using ClustalW [40] with default settings. This first sequence-based alignment was unable to predict the catalytic His residue, however the structural model (described below) predicted His 384 in this position. Subsequent to confirmation of the active site His 384 by mutagenesis, the ClustalW alignment was merged with sequence alignment produced during modeling in SWISS-MODEL [41], as this had more accurately aligned the C-terminal region of the protein, correctly predicting this catalytic residue.

The merged alignment was edited manually to give the final multiple sequence alignment shown in S1 Fig.

A phylogenetic tree was built from the above alignment with the addition of the feruloyl esterase Est1E sequence (UniProt D2YW37_9FIRM) from *Butyrivibrio proteoclasticus (B*. *proteoclasticus)* [42] which was selected as an outgroup to the CE15s as it is a close structural homolog to the *H*. *jecorina* and *M*. *thermophile* enzymes. The tree was constructed in MEGA 6.06 [43] using the Maximum Likelihood method based on the JTT matrix-based model [44]. Initial trees for the heuristic search were obtained automatically by applying Neighbor-Join and BioNJ algorithms to a matrix of pairwise distances estimated using a JTT model. The tree with a topology giving the highest log likelihood value (-20495.6874) was used. There were a total of 525 positions in the final dataset.

The genomic context of putative *ce15* genes were investigated for several bacteria identified from the BLAST taxonomy report as having products with high identity to MZ0003. The complete genomes of *A*. *shahii* WAL 8301, (NC_021030), *R*. *baltica* SH 1 (NC_005027), *D*. *fermentans* DSM 18053 (NC_013037), *P*. *maris* DSM 8797 (ABCE01000018) and *B*. *marina* DSM 3645 (AANZ01000001) were downloaded from NCBI Nucleotide (http://www.ncbi.nlm.nih.gov/nuccore) and viewed using the Artemis genome browser. Gene annotations were directly transferred from the genomes.

### Protein expression and purification

Recombinant MZ0003 was expressed in *E*. *coli* Rosetta DE3 pLys (Novagen, Madison, WI, USA) as a His-tagged maltose binding protein (MBP) fusion protein (His-MBP-MZ0003) from the pCold-II-MBP-TEV vector as described in [18]. Briefly, large scale (5 L) cultivations were carried out in LB medium (100 **μ**g ml^-1^ ampicillin and 34 **μ**g ml^-1^ chloramphenicol). Cells were grown at 37°C until log phase when expression was induced by addition of 0.4 mM IPTG and a reduction in temperature to 15°C. Cells were cultivated for a further for 22 h before harvesting. All subsequent steps were carried out at 4°C. Cells were lysed by French press (one passage at 18,000 psi) in lysis buffer (50 mM Tris HCl pH 8.0; 750 mM NaCl; 5% glycerol; 10 mM MgCl_2_). Cell debris were cleared by centrifugation at 17,000 RCF for 40 min and DNA was removed by treatment with the non-specific nuclease HL-SAN (Arcticzymes, Tromsø, Norway) at 1 unit per mg cell pellet for 1 h. Imidazole was added to the soluble fraction to a final concentration of 20 mM, and His-MBP-MZ0003 was loaded on HisTrap HP 5 ml column (GE Healthcare), washed with 5 column volumes of buffer A (50 mM Tris pH 8.0; 20 mM imidazole; 5% glycerol; 750 mM NaCl) before elution on a linear gradient of 5 column volumes from 0% to 100% Buffer B (50 mM Tris pH 8.0, 750 mM NaCl, 5% glycerol, 500 mM imidazole) and buffer exchange into Buffer C (50 mM Tris pH 8.0, 200 mM NaCl, 500 mM EDTA, 5% glycerol) using a HiPrep 26/10 column (GE Healthcare). The MBP-His tag was cleaved from MZ0003 by incubation with 0.1 mg ml^-1^ Tobacco Etch Virus protease (TEV) overnight. TEV-treated His-MBP-MZ0003 was re-applied to the HisTrap column equilibrated in Buffer C, and the tag-free MZ0003 fraction was eluted in buffer D (50 mM Tris HCl pH 8.0; 5% glycerol; 750 mM NaCl). MZ0003 was purified further by gel filtration on a Superdex HiLoad 16/60 gel filtration column (GE Healthcare, Little Chalfont, UK) in buffer D before up-concentrating to to 2.5 mg ml^-1^ and storage in 5% glycerol at -20°C. The protein concentration was determined by absorbance at 280 nm using a NanoDrop ND-1000 Spectrophotometer (Thermo Scientific, Waltham, MA, USA) using the theoretical molar extinction coefficient 83,225 M^-1^ cm^-1^ based on the MZ0003 amino acid sequence.

Expression of MZ0003 was verified by quadrupole-time of flight liquid chromatography–mass spectrometry (Q-ToF LC–MS/MS). Native PAGE was carried out using the Novex 4–16% Bis-Tris gel system with NativeMark unstained protein standard (Thermo Scientific). SDS–PAGE used Mini-PROTEAN TGX precast gels (Bio-Rad Laboratories) and Mark12 molecular weight marker (Thermo Scientific).

### Determination of Glucuronoyl esterase activity by TLC

Glucuronoyl esterase activity was assayed by Thin Layer Chromatography (TLC) following a modified protocol of [8] and using the substrates methyl 4-O-methyl-D-glucopyranosyluronate (Institute of Chemistry, Slovak Academy of Sciences) dissolved in 100% methanol, allyl D-glucuronate, benzyl D-glucuronate and methyl D-glucuronate (Carbosynth Limited, UK) dissolved in water. Enzyme reactions were performed by incubating 50**μ**g of purified enzyme with 8 mM of substrate in 50 mM sodium phosphate buffer, pH 6.0 with a final volume of 100 **μ**l, at 25°C for 90 min. The qualitative experiment was performed on Silica gel 60 TLC plates (Merck) developed in CH_2_Cl_2_/CH_3_OH/H_2_O (80:25:4) and visualized with 40% of sulfuric acid and 5% of potassium dichromate and heating for 10 min at 100°C.

### Quantitative end point assays for deacetylase (esterase) activity

MZ0003 deacetylase activity was detected using the *Acetate Colorimetric Assay Kit* (Sigma-Aldrich, St Louis, MO, USA). The substrates used, purchased from Sigma-Aldrich, were: 4-methylumbelliferyl acetate and 7-aminocephalosporanic acid (dissolved in DMSO); α-naphtyl acetate, β-D-glucose pentaacetate and β-D-galactose pentaacetate (dissolved in methanol); 5-bromo-4-chloro-3-indoyl acetate (dissolved in ethanol); *p-*NP acetate and cellulose acetate (dissolved in acetonitrile). The reaction mixtures, each containing 10 **μ**g of protein and substrate, were pre-incubated in 5 mM Na-phosphate buffer pH 7.3 for 30 min at 35°C. Thereafter the assay was developed according to the manufacturer’s instructions and the absorbance was read at 450 nm. Values for each substrate were taken as the mean of three independent measurements after subtraction of a blank sample, without MZ0003, to correct for background degradation. The amount of acetate liberated was calculated in nM **μ**l^-1^ from a standard curve according to the manufacturer’s instructions.

### Time-resolved assay with p-NP esters

Standard conditions: unless otherwise indicated, MZ0003 activity was measured at 405 nm for 20 minutes in a spectrophotometer (SpectraMax Me2, Microplate reader, Molecular Devices, Sunnyvale, CA, USA). Reaction mixture (100 **μ**l) contained 3.5 **μ**g of enzyme, 0.1 M Tris-HCl buffer (pH 8.0) and 1 mM *p*-NP acetate (Sigma) as substrate dissolved in 100% acetonitrile. Enzymatic activity was assayed in triplicate with an appropriate blank for the correction of the auto hydrolysis of the substrate. One unit of enzymatic activity was defined as the amount of protein that released 1 **μ**mol of p-nitrophenoxide/min from *p*-NP esters. Molecular coefficient extinction of 18,000 M^-1^ cm^-1^ was used for the calculation.

MZ0003 esterase activity was determined towards the acyl chain length of different *p*-NP esters, dissolved in 100% acetonitrile and purchased from Sigma-Aldrich: *p*-NP acetate, *p*-NP butyrate and *p*-NP octanoate, in the standard assay conditions mentioned in the enzyme activity assay paragraph.

Kinetic parameters were calculated for *p*-NP acetate using a concentration range of 0 to 4.5 mM in presence of 10 **μ**g of enzyme and 0.1 M Tris-HCl pH 8.0. Initial velocities were calculated with the SoftMax Pro software (Molecular Devices) and then data were fitted with the enzyme kinetic analysis module of Sigmaplot 12.5 (Systat Software, Inc., San Jose, CA).

### pH and temperature dependence assays

The effect of pH on enzyme activity was estimated by varying the pH from 5.0 to 10.5 in 0.1 M buffers at 30°C. MES buffer was used for pH 5.0–6.0, Na-phosphate buffer from pH 6.0–7.5, Tris-HCl buffer for pH 7.5–9.5 and CAPS buffer for pH 9.5–10.5. The esterase activity was measured at 348 nm, the pH-independent isosbestic point of *p*-nitrophenol and the *p*-nitrophenoxide ion. To define the temperature profiling, the activity was evaluated in triplicate over temperature range from 10 to 50°C. Percentage of enzyme activity was normalized to the highest activity detected in the pH or temperature range. For both pH and temperature profiles two sets of measurements were made with either 1 M NaCl, or no additional salt.

### Temperature stability assays

Thermostability was measured at 35°C, in presence of 1 M NaCl, by evaluating residual activity in triplicate after incubation of the enzyme for 120 min with 20 min intervals at various temperatures: 10, 20, 30 and 40°C. 100% of the enzyme activity was considered as the activity of enzyme without incubation.

### Additive effect assays

MZ0003 activity was evaluated in presence of: ethylenediaminetetraacetic acid (EDTA), urea, 2-mercaptoethanol (2-MeOH), Sodium dodecyl sulfate (SDS), phenylmethanesulfonyl fluoride (PMSF), Dithiothreitol (DTT) and different monovalent and divalent metals: KCl, LiCl, AgCl_2_, NaF, ZnCl_2_, CaCl_2_, MgCl_2_, CoCl_2_, SnCl_2_, CuCl_2_, MnCl_2_ and CdCl_2_. Chemical reagents or metals at a final concentration of 1 mM and 10 mM were incubated with the enzyme in 0.1 M Tris-HCl pH 8.0 for 1 hour on ice. Substrate was then added to the reaction mixture and the assay was performed in triplicate at 35°C for 20 minutes using appropriate blanks as controls. Percentage relative activity was reported, considering 100% activity in the absence of additives or metals.

### Differential Scanning Calorimetry and Differential Scanning fluorimetry

Thermal unfolding of MZ0003 was studied by DSC using a Nano-Differential scanning Calorimeter III (Calorimetry Sciences Corporation, Lindon, USA). Protein was dialyzed for 8 h at 4°C against 50 mM HEPES pH 8.0, 750 mM NaCl and then degassed for 15 min. DSC experiments were performed at a scan rate of 1°C min^-1^ in a range of 5 to 75°C and the dialysis buffer was used as reference buffer in the runs. The NanoAnalyze software was used to calculate the melting temperature (T_m_ value) by the subtraction of the buffer-buffer baseline from the protein scan and fitting the data to a two-state model.

The T_m_ value of the protein melting curve was also assessed by DSF as described in using the Biorad MiniOpticon real-time PCR instrument in a range from 10 to 80°C. SYPRO® Orange Dye (Sigma-Aldrich) was added at a final concentration of 0.6% to 3 **μ**g of protein and 50 mM HEPES pH 8.0 varying the NaCl concentrations from 0 to 2.5 M in a final volume of 25 **μ**l.

### Homology modeling

A structural model of MZ0003 was built using the SWISS-MODEL server (http://swissmodel.expasy.org/) in automated mode. The mature MZ0003 sequence was used as a search query, and three models were built using the most homologous sequences as templates; the two CE15 structures 3pic from *H*. *jecorina* and 4G4G from *M*. *thermophila* StGE2 (25.62% and 23.17% identity respectively) and the Esterase APC40077 3S8Y of *Oleispira Antarctica* (16.80%). Both CE15 templates produced very similar predictions, and the model based on 4G4G was selected for further analysis based on its high resolution.

### Site-direct mutagenesis

Site-direct mutagenesis method was used to generate four different MZ0003 mutants by using the QuickChange Site-Directed Mutagenesis kit (Agilent Technologies) according to the manufacturers’ instructions. Mutagenic primers with a single or double amino acid substitutions were: MZ3_K127A_E128A_BK: 5'-CGACTCGGTCAACCAGATTGCTGCGTCCTTGTGGATGGTGTGG -3'; MZ3_K127A_E128A_FD: 5'- CCACACCATCCACAAGGACGCAGCAATCTGGTTGACCGAGTCG-3'; MZ3_S243A_BK: 5'-CCAATCGCGCATGGCCCAGCACCGC-3'; MZ3_S243A_FD: 5'-GCGGTGCTGGGCCATGCGCGATTGG-3'; MZ3_C266A_BK: 5'-GCCGCCACAACCTGAAGCGTTCGAGATCGTCAGC-3'; MZ3_C266A_FD: 5'-GCTGACGATCTCGAACGCTTCAGGTTGTGGCGGC-3'; MZ3_H384A_BK: 5'-GCGGTGACGTCGGCCTTGCCGGGCCG-3'; MZ3_H384A_FD: 5'-CGGCCCGGCAAGGCCGACGTCACCGC-3' (modified codons are underlined). Mutations were confirmed by sequence analysis of both DNA strands and purified mutant proteins were tested for activity under standard assay conditions.

## Supporting Information

S1 Fig Multiple sequence alignment of characterised CE15 enzymes. Bacterial homologs are boxed in blue. The secondary structural elements of the *M*. *thermophila* StGE2 crystal structure (PDB 4g4g) are given above the alignment, and numbering pertains to this sequence. Secondary structural elements predicted from homology modelling of MZ0003 are given below the sequences. Confirmed active site residues of StGE2are indicated by red arrows above the sequence, while catalytic residues identified by mutagenesis in MZ0003 are indicated in green on the lower sequence. The residues which were mutated without effect in MZ0003 is indicated with a grey arrow. Insertions in the MZ0003 relative to StGE2 are shaded yellow. The sequences have been truncated relative to position 44 of StGE2 which removes the secretion signal predicted for most of the bacterial enzymes as well as the long N-terminal extension of *B*. *xylanisolvens* CE15 and the N-terminal domains of *R*. *flavefaciens* CesA.
(TIF)

S2 Fig SDS-PAGE analysis of MZ0003. Lane1: soluble fraction. Lane 2: purified protein. Lane 3: Mark12 molecular weight marker. Native-PAGE analysis of MZ0003 purified protein. Lane 1: NativeMark unstained protein standard; Lane 2: (10 μg) MZ0003; Lane 3: (5 μg) MZ0003; Lane 4: (1 μg) MZ0003; Lane 5: Conalbumin, marker protein.
(TIF)

S3 Fig TLC-based assay for hydrolysis of methyl 4-O-methyl-D-glucopyranosyluronate by the general carboxyesterase ThaEst2349 (tha). MZ0003 is used as a positive control (mz3) and no added enzyme as the negative control (neg). Assay conditions are as given in the manuscript, and time points were taken at 1.5, 5 and 18 hours.
(TIF)

S4 Fig Michelis-Menten saturation curve for MZ0003. Kinetic parameters calculated using *p*-NP acetate (0 to 4.5mM) with 10 μg of enzyme. Buffer is 0.1 M Tris-HCl pH 8.0 with no additional salt **(A)** and 1 M NaCl **(B)**. All data are the average of three independent experiments, error is the standard deviation.
(TIF)

S5 Fig Quality of MZ0003 structural model. **A**) Two views of the structural model of MZ0003 based on the template 4G4G. Structures are coloured by QMEAN4 score, with best scoring regions in blue and worst scoring regions in orange. Structural elements having no counterpart in the 4G4G template are indicated with green arrows and numbered 1–4. **B)** Sequence alignment between MZ0003 and 4G4G. Non-aligned regions are indicated by green boxes with numbering corresponding to structural elements indicated in A) **C)** Local quality estimate of the MZ0003 model plotted against residue number. Sequence regions 1–4 correspond to A and B.
(TIF)

S1 Table Homologs to MZ0003 used for sequence alignment and phylogeny construction (DOCX)

## Abbreviations

2-MeOH: 2-Mercaptoethanol
HEPES: 4-(2-Hydroxyethyl)-1-piperazineethanesulfonic acid
MeGlcA: 4-O-methyl-D-glucuronic acid
BLAST: Basic Local Alignment Search Tool
CE15: Carbohydrate Esterase 15
CAZymes: Carbohydrate-Active enzymes
DSC: Differential Scanning Calorimetry
DSF: Differential Scanning Fluorimetry
DTT: Dithiothreitol
EDTA: Ethylenediaminetetraacetic acid
GE: Glucuronoyl Esterases
IPTG: Isopropyl β-D-1-thiogalactopyranoside
LCCs: Lignin-Carbohydrate Complexes
MBP: Maltose Binding Protein
CAPS: N-cyclohexyl-3-aminopropanesulfonic acid
PMSF: Phenylmethanesulfonyl Fluoride
*p*-NP-acetate: *p*-Nitrophenyl acetate
PAGE: Poly Acrylamide Gel Electrophoresis
RCF: Relative Centrifugal Force
SDS: Sodium dodecyl sulfate
TLC: Thin Layer Chromatography
TEV: Tobacco Etch Virus
Tris-HCl: Trizma HCl
