# Supplementary figures and images for: Biochemical Characterization of a Family 15 Carbohydrate Esterase from a Bacterial Marine Arctic Metagenome
Source: PLoS One. 2016 Jul 19;11(7):e0159345. doi: 10.1371/journal.pone.0159345 (PMC4951047; doi:10.1371/journal.pone.0159345)

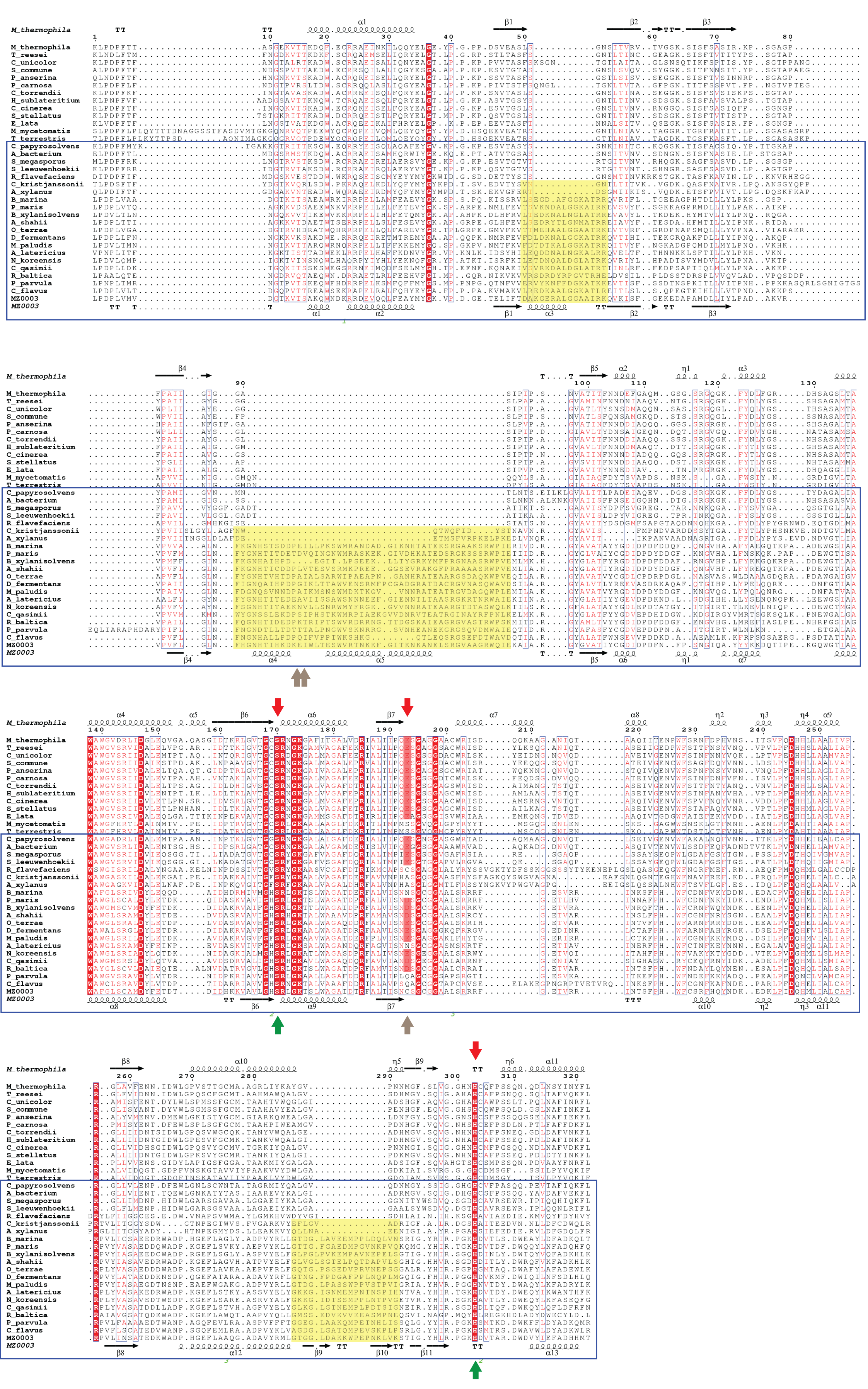

Supplement: S1 Fig — Bacterial homologs are boxed in blue. The secondary structural elements of the M. thermophila StGE2 crystal structure (PDB 4g4g) are given above the alignment, and numbering pertains to this sequence. Secondary structural elements predicted from homology modelling of MZ0003 are given below the sequences. Confirmed active site residues of StGE2are indicated by red arrows above the sequence, while catalytic residues identified by mutagenesis in MZ0003 are indicated in green on the lower sequence. The residues which were mutated without effect in MZ0003 is indicated with a grey arrow. Insertions in the MZ0003 relative to StGE2 are shaded yellow. The sequences have been truncated relative to position 44 of StGE2 which removes the secretion signal predicted for most of the bacterial enzymes as well as the long N-terminal extension of B. xylanisolvens CE15 and the N-terminal domains of R. flavefaciens CesA. (TIF) [file pone.0159345.s001.tif]

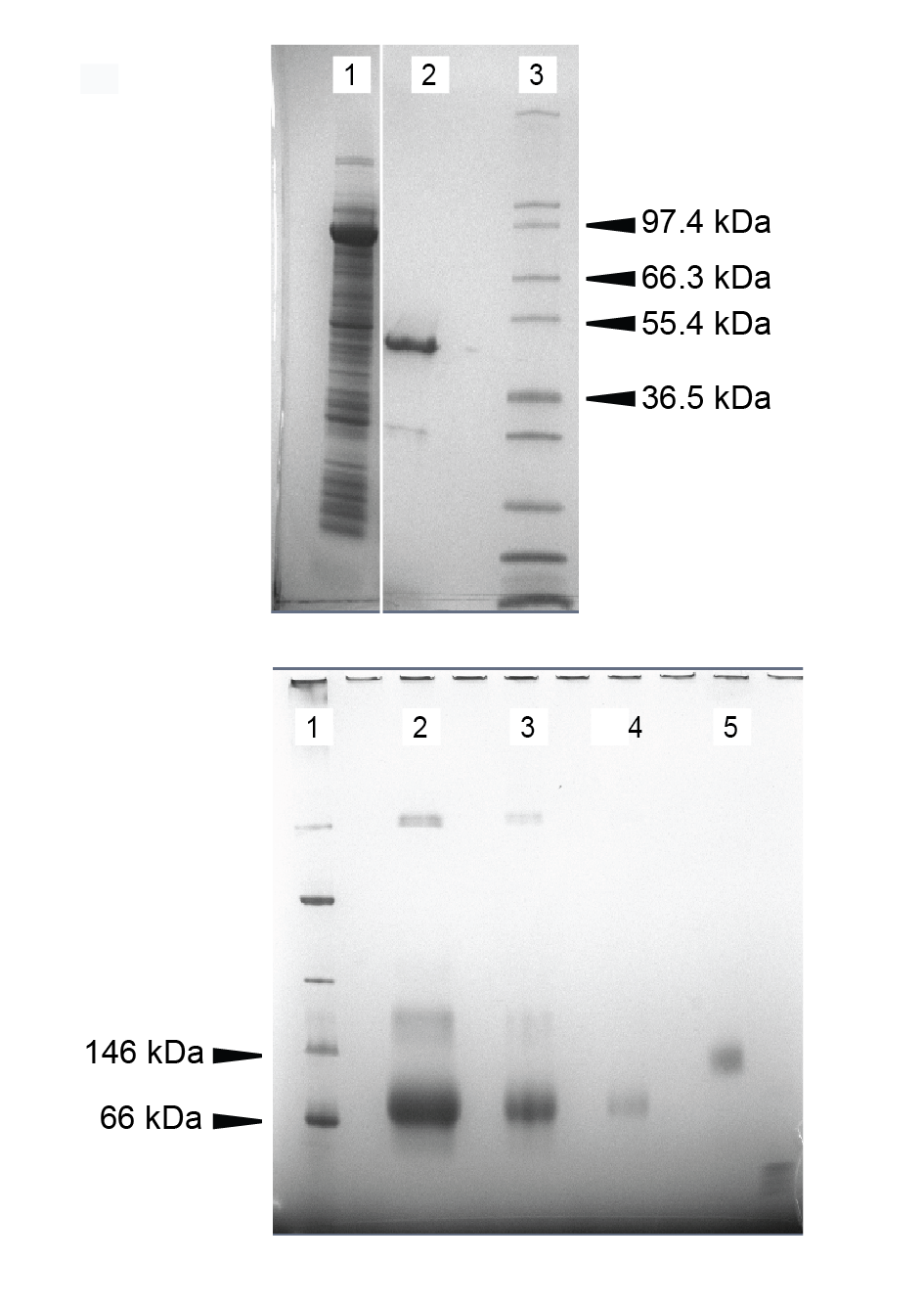

Supplement: S2 Fig — Lane1: soluble fraction. Lane 2: purified protein. Lane 3: Mark12 molecular weight marker. Native-PAGE analysis of MZ0003 purified protein. Lane 1: NativeMark unstained protein standard; Lane 2: (10 μg) MZ0003; Lane 3: (5 μg) MZ0003; Lane 4: (1 μg) MZ0003; Lane 5: Conalbumin, marker protein. (TIF) [file pone.0159345.s002.tif]

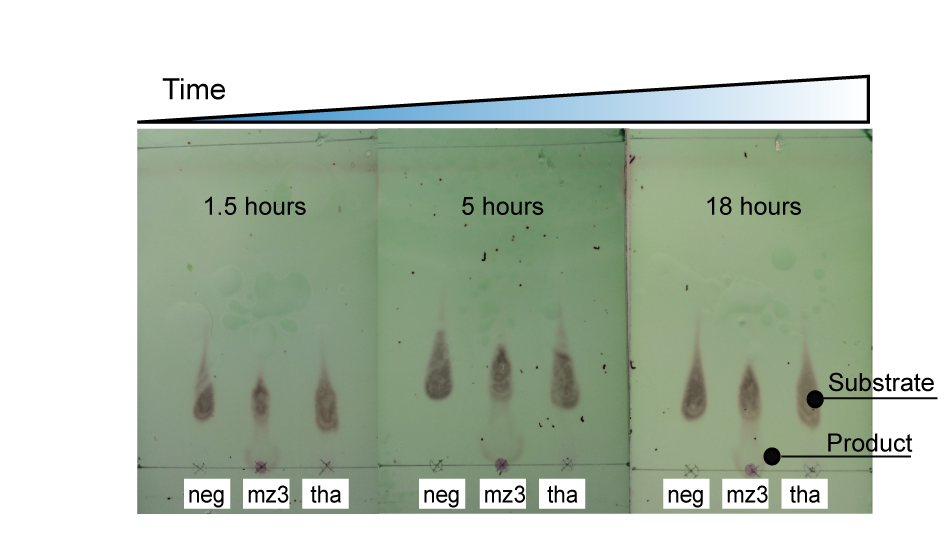

Supplement: S3 Fig — MZ0003 is used as a positive control (mz3) and no added enzyme as the negative control (neg). Assay conditions are as given in the manuscript, and time points were taken at 1.5, 5 and 18 hours. (TIF) [file pone.0159345.s003.tif]

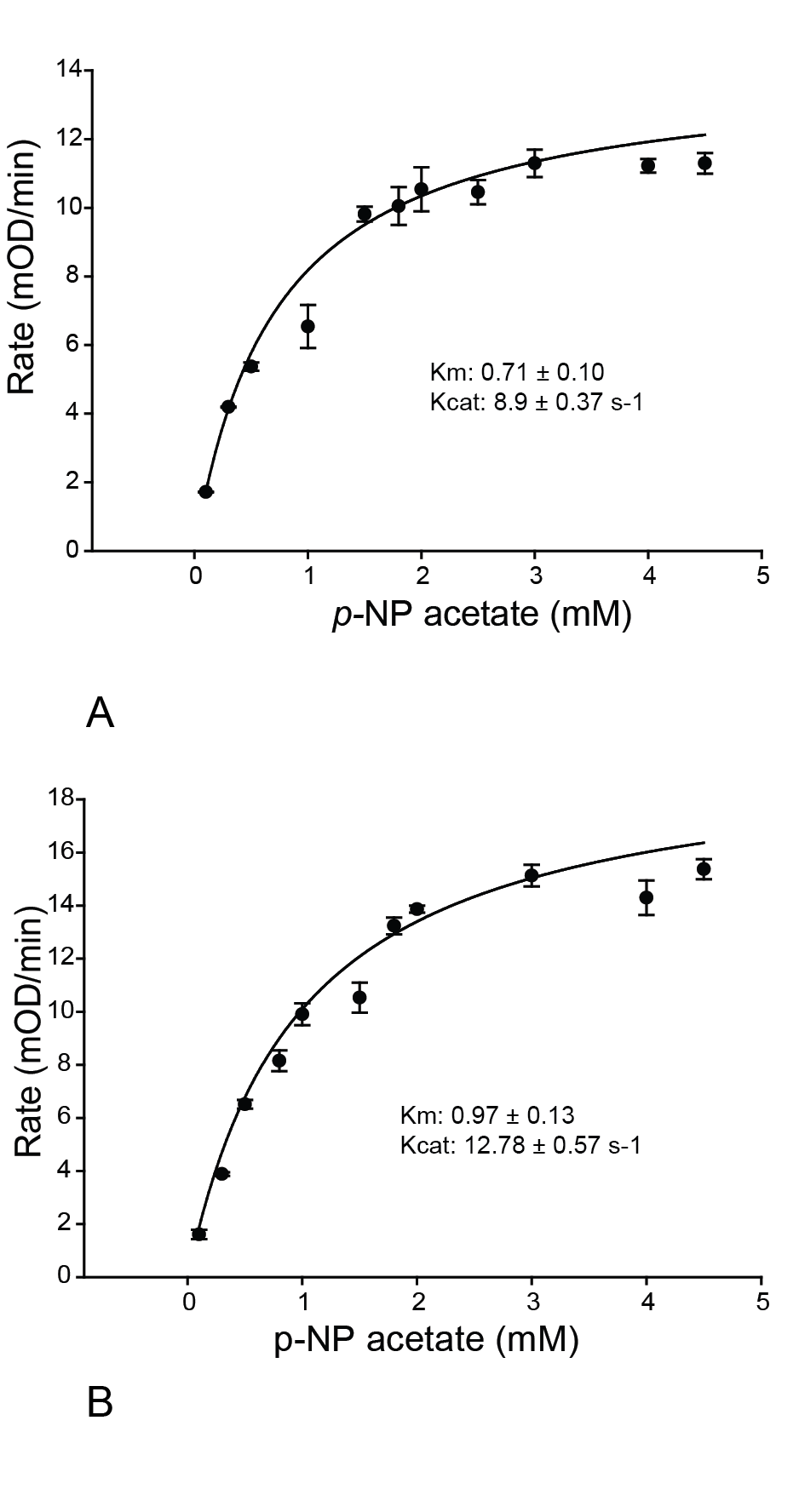

Supplement: S4 Fig — Kinetic parameters calculated using p-NP acetate (0 to 4.5mM) with 10 μg of enzyme. Buffer is 0.1 M Tris-HCl pH 8.0 with no additional salt (A) and 1 M NaCl (B). All data are the average of three independent experiments, error is the standard deviation. (TIF) [file pone.0159345.s004.tif]

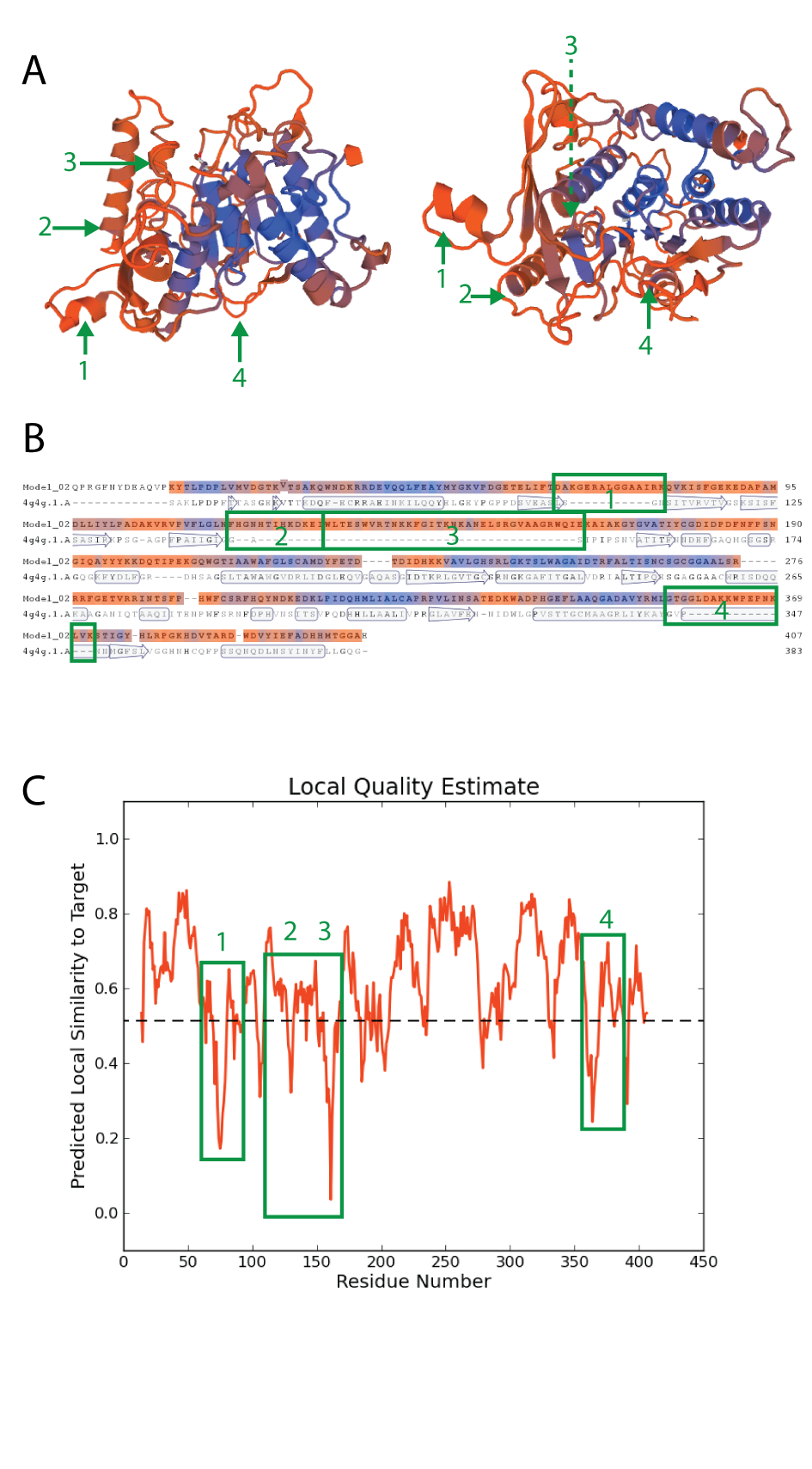

Supplement: S5 Fig — A) Two views of the structural model of MZ0003 based on the template 4G4G. Structures are coloured by QMEAN4 score, with best scoring regions in blue and worst scoring regions in orange. Structural elements having no counterpart in the 4G4G template are indicated with green arrows and numbered 1–4. B) Sequence alignment between MZ0003 and 4G4G. Non-aligned regions are indicated by green boxes with numbering corresponding to structural elements indicated in A) C) Local quality estimate of the MZ0003 model plotted against residue number. Sequence regions 1–4 correspond to A and B. (TIF) [file pone.0159345.s005.tif]
