# Supplementary material for: Biochemical Characterization of a Family 15 Carbohydrate Esterase from a Bacterial Marine Arctic Metagenome
Source: PLoS One. 2016 Jul 19;11(7):e0159345. doi: 10.1371/journal.pone.0159345 (PMC4951047; doi:10.1371/journal.pone.0159345)
Supplement: S1 Table — (DOCX) [file pone.0159345.s006.docx]

| **Classification** | **Phylum** | **Species** | **Habitat** | **Gene identifier** | **Reference ^a^** |
| --- | --- | --- | --- | --- | --- |
| Bacteria | Planctomycetes | *Blastopirellula marina DSM 3645* | Marine | gi\|87290827 | (Woebken, Teeling et al. 2007) |
|  |  | *Planctomyces maris DSM 8797* | Marine | gi\|148844708 | (Woebken, Teeling et al. 2007) |
|  |  | Rhodopirellula baltica SH 1 | Marine | gi\|32444102 | (Glockner, Kube et al. 2003) |
|  | Bacteroidetes | Bacteroides xylanisolvens CL03T12C04 | Human Microbiome | gi\|392693200 | BioProject ID: PRJNA64839 |
|  |  | *Alistipes shahii WAL 8301* | Human intestine | gi\|479186408 | (Song, Kononen et al. 2006) |
|  |  | *Dyadobacter fermentans DSM 18053* | Plant tissue | gi\|255039446 | (Lang, Lapidus et al. 2009) |
|  |  | *Mucilaginibacter paludis DSM 18603* | Peat bog | gi\|373894621 | (Pankratov, Tindall et al. 2007) |
|  |  | *Arenibacter latericius* DSM 15913 | Marine sediment | gi\|652414843 | BioProject ID: 188327 |
|  |  | *Niastella koreensis GR20-10* | Soil | gi\|375145395 | BioProject ID: 42711 |
|  |  | Cyclobacterium qasimii M12-11B | Marine Arctic | gi\|523602036 | (Shivaji, Ara et al. 2013) |
|  | Verrucomicrobia | *Opitutus terrae PB90-1* | Rice paddy | gi\|182411941 | (van Passel, Kant et al. 2011) |
|  |  | Pedosphaera parvula Ellin514 | Soil | gi\|223894191 | (Kant, van Passel et al. 2011) |
|  |  | *Chthoniobacter flavus Ellin428* | Soil | gi\|196224705 | (Kant, van Passel et al. 2011) |
|  | Proteobacteria; | *Alteromonadaceae bacterium Bs12* | Marine Symbiont of shipworm | gi\|919136150 | BioProject ID: 195847 |
|  | Firmicutes | *Ruminococcus flavefaciens* | Cow rumen | gi\|5834678 | (Aurilia, Martin et al. 2000) |
|  |  | *Caldicellulosiruptor kristjanssonii* I77R1B | hot spring biomat | gi\|312181537 | (Blumer-Schuette, Ozdemir et al. 2011) |
|  |  | *Amphibacillus xylanus* NBRC 15112 | Compost | gi\|504823370 | (Niimura, Koh et al. 1990) |
|  |  | *Clostridium* papyrosolvens C7 | Anerobic freshwater mud | gi\|523698682 | (Zepeda, Dassa et al. 2013) |
|  | Actinobacteria | *Streptomyces leeuwenhoekii* DSM:42122 | Hyper-arid desert soil | gi\|822876275 | (Gomez-Escribano, Castro et al. 2015) |
| Fungi | Ascomycota | *Trichoderma reesei (synonym Hypocrea jecorina)* | Wood-rotting | gi\|31747154 | (Li, Spanikova et al. 2007) |
|  |  | *Podospora anserina* | Wood-rotting | gi\|170936249 | (Katsimpouras, Benarouche et al. 2014) |
|  |  | *Myceliophthora thermophila (Sporotrichum thermophile*) | Soil and compost | gi\|408407612 | (Topakas, Moukouli et al. 2010) |
|  |  | *Madurella mycetomatis* | Human pathogen | gi\|923155338 | (Ahmed, van Leeuwen et al. 2004) |
|  |  | *Thielavia terrestris* NRRL 8126 | Wood-rotting | gi\|367051553 | (Berka, Grigoriev et al. 2011) |
|  |  | *Eutypa lata* UCREL1 | Plant pathogen | gi\|629654525 | (Rolshausen, Greve et al. 2008) |
|  | Basidiomycota | *Phanerochaete carnosa* | Wood-rotting |  | (Duranova, Hirsch et al. 2009) |
|  |  | *Cerrena unicolor* | Wood-rotting | gi\|72842450 | (d'Errico, Jørgensen et al. 2015) |
|  |  | *Schizophyllum commune* | Wood-rotting | gi\|302673204 | (Spanikova and Biely 2006) |
|  |  | *Sphaerobolus stellatus* SS14 | Wood-rotting | gi\|749878959 | (Kohler, Kuo et al. 2015) |
|  |  | *Coprinopsis cinerea* okayama7#130 | Wood-rotting | gi\|169856114 | (Stajich, Wilke et al. 2010) |
|  |  | *Hypholoma sublateritium* FD-334 SS-4 | Wood-rotting | gi\|763732596 | (Kohler, Kuo et al. 2015) |
|  |  | *Cylindrobasidium torrendii* FP15055 ss-10 | Wood-rotting | gi\|761946376 | (Floudas, Held et al. 2015) |
|  |  |  |  |  |  |

**a. References:**

Ahmed, A. O., W. van Leeuwen, A. Fahal, W. van de Sande, H. Verbrugh and A. van Belkum (2004). "Mycetoma caused by Madurella mycetomatis: a neglected infectious burden." Lancet Infect Dis **4**(9): 566-574.

Aurilia, V., J. C. Martin, S. I. McCrae, K. P. Scott, M. T. Rincon and H. J. Flint (2000). "Three multidomain esterases from the cellulolytic rumen anaerobe Ruminococcus flavefaciens 17 that carry divergent dockerin sequences." Microbiology **146 ( Pt 6)**: 1391-1397.

Berka, R. M., I. V. Grigoriev, R. Otillar, A. Salamov, J. Grimwood, I. Reid, N. Ishmael, T. John, C. Darmond, M.-C. Moisan, B. Henrissat, P. M. Coutinho, V. Lombard, D. O. Natvig, E. Lindquist, J. Schmutz, S. Lucas, P. Harris, J. Powlowski, A. Bellemare, D. Taylor, G. Butler, R. P. de Vries, I. E. Allijn, J. van den Brink, S. Ushinsky, R. Storms, A. J. Powell, I. T. Paulsen, L. D. H. Elbourne, S. E. Baker, J. Magnuson, S. LaBoissiere, A. J. Clutterbuck, D. Martinez, M. Wogulis, A. L. de Leon, M. W. Rey and A. Tsang (2011). "Comparative genomic analysis of the thermophilic biomass-degrading fungi Myceliophthora thermophila and Thielavia terrestris." Nat Biotech **29**(10): 922-927.

Blumer-Schuette, S. E., I. Ozdemir, D. Mistry, S. Lucas, A. Lapidus, J. F. Cheng, L. A. Goodwin, S. Pitluck, M. L. Land, L. J. Hauser, T. Woyke, N. Mikhailova, A. Pati, N. C. Kyrpides, N. Ivanova, J. C. Detter, K. Walston-Davenport, S. Han, M. W. Adams and R. M. Kelly (2011). "Complete genome sequences for the anaerobic, extremely thermophilic plant biomass-degrading bacteria Caldicellulosiruptor hydrothermalis, Caldicellulosiruptor kristjanssonii, Caldicellulosiruptor kronotskyensis, Caldicellulosiruptor owensensis, and Caldicellulosiruptor lactoaceticus." J Bacteriol **193**(6): 1483-1484.

d'Errico, C., J. O. Jørgensen, K. B. R. M. Krogh, N. Spodsberg, R. Madsen and R. N. Monrad (2015). "Enzymatic degradation of lignin-carbohydrate complexes (LCCs): Model studies using a fungal glucuronoyl esterase from Cerrena unicolor." Biotechnology and Bioengineering: n/a-n/a.

Duranova, M., J. Hirsch, K. Kolenova and P. Biely (2009). "Fungal glucuronoyl esterases and substrate uronic acid recognition." Biosci Biotechnol Biochem **73**(11): 2483-2487.

Floudas, D., B. W. Held, R. Riley, L. G. Nagy, G. Koehler, A. S. Ransdell, H. Younus, J. Chow, J. Chiniquy, A. Lipzen, A. Tritt, H. Sun, S. Haridas, K. LaButti, R. A. Ohm, U. Kües, R. A. Blanchette, I. V. Grigoriev, R. E. Minto and D. S. Hibbett (2015). "Evolution of novel wood decay mechanisms in Agaricales revealed by the genome sequences of Fistulina hepatica and Cylindrobasidium torrendii." Fungal Genetics and Biology **76**: 78-92.

Glockner, F. O., M. Kube, M. Bauer, H. Teeling, T. Lombardot, W. Ludwig, D. Gade, A. Beck, K. Borzym, K. Heitmann, R. Rabus, H. Schlesner, R. Amann and R. Reinhardt (2003). "Complete genome sequence of the marine planctomycete Pirellula sp. strain 1." Proc Natl Acad Sci U S A **100**(14): 8298-8303.

Gomez-Escribano, J. P., J. F. Castro, V. Razmilic, G. Chandra, B. Andrews, J. A. Asenjo and M. J. Bibb (2015). "The Streptomyces leeuwenhoekii genome: de novo sequencing and assembly in single contigs of the chromosome, circular plasmid pSLE1 and linear plasmid pSLE2." BMC Genomics **16**(1): 1-11.

Kant, R., M. W. van Passel, A. Palva, S. Lucas, A. Lapidus, T. Glavina del Rio, E. Dalin, H. Tice, D. Bruce, L. Goodwin, S. Pitluck, F. W. Larimer, M. L. Land, L. Hauser, P. Sangwan, W. M. de Vos, P. H. Janssen and H. Smidt (2011). "Genome sequence of Chthoniobacter flavus Ellin428, an aerobic heterotrophic soil bacterium." J Bacteriol **193**(11): 2902-2903.

Kant, R., M. W. van Passel, P. Sangwan, A. Palva, S. Lucas, A. Copeland, A. Lapidus, T. Glavina del Rio, E. Dalin, H. Tice, D. Bruce, L. Goodwin, S. Pitluck, O. Chertkov, F. W. Larimer, M. L. Land, L. Hauser, T. S. Brettin, J. C. Detter, S. Han, W. M. de Vos, P. H. Janssen and H. Smidt (2011). "Genome sequence of "Pedosphaera parvula" Ellin514, an aerobic Verrucomicrobial isolate from pasture soil." J Bacteriol **193**(11): 2900-2901.

Katsimpouras, C., A. Benarouche, D. Navarro, M. Karpusas, M. Dimarogona, J. G. Berrin, P. Christakopoulos and E. Topakas (2014). "Enzymatic synthesis of model substrates recognized by glucuronoyl esterases from Podospora anserina and Myceliophthora thermophila." Appl Microbiol Biotechnol **98**(12): 5507-5516.

Kohler, A., A. Kuo, L. G. Nagy, E. Morin, K. W. Barry, F. Buscot, B. Canback, C. Choi, N. Cichocki, A. Clum, J. Colpaert, A. Copeland, M. D. Costa, J. Dore, D. Floudas, G. Gay, M. Girlanda, B. Henrissat, S. Herrmann, J. Hess, N. Hogberg, T. Johansson, H. R. Khouja, K. LaButti, U. Lahrmann, A. Levasseur, E. A. Lindquist, A. Lipzen, R. Marmeisse, E. Martino, C. Murat, C. Y. Ngan, U. Nehls, J. M. Plett, A. Pringle, R. A. Ohm, S. Perotto, M. Peter, R. Riley, F. Rineau, J. Ruytinx, A. Salamov, F. Shah, H. Sun, M. Tarkka, A. Tritt, C. Veneault-Fourrey, A. Zuccaro, A. Tunlid, I. V. Grigoriev, D. S. Hibbett and F. Martin (2015). "Convergent losses of decay mechanisms and rapid turnover of symbiosis genes in mycorrhizal mutualists." Nat Genet **47**(4): 410-415.

Lang, E., A. Lapidus, O. Chertkov, T. Brettin, J. C. Detter, C. Han, A. Copeland, T. Glavina Del Rio, M. Nolan, F. Chen, S. Lucas, H. Tice, J. F. Cheng, M. Land, L. Hauser, Y. J. Chang, C. D. Jeffries, M. Kopitz, D. Bruce, L. Goodwin, S. Pitluck, G. Ovchinnikova, A. Pati, N. Ivanova, K. Mavrommatis, A. Chen, K. Palaniappan, P. Chain, J. Bristow, J. A. Eisen, V. Markowitz, P. Hugenholtz, M. Goker, M. Rohde, N. C. Kyrpides and H. P. Klenk (2009). "Complete genome sequence of Dyadobacter fermentans type strain (NS114)." Stand Genomic Sci **1**(2): 133-140.

Li, X. L., S. Spanikova, R. P. de Vries and P. Biely (2007). "Identification of genes encoding microbial glucuronoyl esterases." FEBS Lett **581**(21): 4029-4035.

Niimura, Y., E. Koh, F. Yanagida, K.-I. Suzuki, K. Komagata and M. Kozaki (1990). "Amphibacillus xylanus gen. nov., sp. nov., a Facultatively Anaerobic Sporeforming Xylan-Digesting Bacterium Which Lacks Cytochrome, Quinone, and Catalase." International Journal of Systematic and Evolutionary Microbiology **40**(3): 297-301.

Pankratov, T. A., B. J. Tindall, W. Liesack and S. N. Dedysh (2007). "Mucilaginibacter paludis gen. nov., sp. nov. and Mucilaginibacter gracilis sp. nov., pectin-, xylan- and laminarin-degrading members of the family Sphingobacteriaceae from acidic Sphagnum peat bog." Int J Syst Evol Microbiol **57**(Pt 10): 2349-2354.

Rolshausen, P. E., L. C. Greve, J. M. Labavitch, N. E. Mahoney, R. J. Molyneux and W. D. Gubler (2008). "Pathogenesis of Eutypa lata in grapevine: identification of virulence factors and biochemical characterization of cordon dieback." Phytopathology **98**(2): 222-229.

Shivaji, S., S. Ara, A. Singh and A. Kumar Pinnaka (2013). "Draft Genome Sequence of Cyclobacterium qasimii Strain M12-11BT, Isolated from Arctic Marine Sediment." Genome Announc **1**(4).

Song, Y., E. Kononen, M. Rautio, C. Liu, A. Bryk, E. Eerola and S. M. Finegold (2006). "Alistipes onderdonkii sp. nov. and Alistipes shahii sp. nov., of human origin." Int J Syst Evol Microbiol **56**(Pt 8): 1985-1990.

Spanikova, S. and P. Biely (2006). "Glucuronoyl esterase--novel carbohydrate esterase produced by Schizophyllum commune." FEBS Lett **580**(19): 4597-4601.

Stajich, J. E., S. K. Wilke, D. Ahren, C. H. Au, B. W. Birren, M. Borodovsky, C. Burns, B. Canback, L. A. Casselton, C. K. Cheng, J. Deng, F. S. Dietrich, D. C. Fargo, M. L. Farman, A. C. Gathman, J. Goldberg, R. Guigo, P. J. Hoegger, J. B. Hooker, A. Huggins, T. Y. James, T. Kamada, S. Kilaru, C. Kodira, U. Kues, D. Kupfer, H. S. Kwan, A. Lomsadze, W. Li, W. W. Lilly, L. J. Ma, A. J. Mackey, G. Manning, F. Martin, H. Muraguchi, D. O. Natvig, H. Palmerini, M. A. Ramesh, C. J. Rehmeyer, B. A. Roe, N. Shenoy, M. Stanke, V. Ter-Hovhannisyan, A. Tunlid, R. Velagapudi, T. J. Vision, Q. Zeng, M. E. Zolan and P. J. Pukkila (2010). "Insights into evolution of multicellular fungi from the assembled chromosomes of the mushroom Coprinopsis cinerea (Coprinus cinereus)." Proc Natl Acad Sci U S A **107**(26): 11889-11894.

Topakas, E., M. Moukouli, M. Dimarogona, C. Vafiadi and P. Christakopoulos (2010). "Functional expression of a thermophilic glucuronoyl esterase from Sporotrichum thermophile: identification of the nucleophilic serine." Applied Microbiology and Biotechnology **87**(5): 1765-1772.

van Passel, M. W., R. Kant, A. Palva, A. Copeland, S. Lucas, A. Lapidus, T. Glavina del Rio, S. Pitluck, E. Goltsman, A. Clum, H. Sun, J. Schmutz, F. W. Larimer, M. L. Land, L. Hauser, N. Kyrpides, N. Mikhailova, P. P. Richardson, P. H. Janssen, W. M. de Vos and H. Smidt (2011). "Genome sequence of the verrucomicrobium Opitutus terrae PB90-1, an abundant inhabitant of rice paddy soil ecosystems." J Bacteriol **193**(9): 2367-2368.

Woebken, D., H. Teeling, P. Wecker, A. Dumitriu, I. Kostadinov, E. F. Delong, R. Amann and F. O. Glockner (2007). "Fosmids of novel marine Planctomycetes from the Namibian and Oregon coast upwelling systems and their cross-comparison with planctomycete genomes." ISME J **1**(5): 419-435.

Zepeda, V., B. Dassa, I. Borovok, R. Lamed, E. A. Bayer and J. H. D. Cate (2013). "Draft Genome Sequence of the Cellulolytic Bacterium Clostridium papyrosolvens C7 (ATCC 700395)." Genome Announcements **1**(5): e00698-00613.
